# Supplementary material for: Rapid optimisation of fragments and hits to lead compounds from screening of crude reaction mixtures
Source: Commun Chem. 2020 Sep 2;3:122. doi: 10.1038/s42004-020-00367-0 (PMC9814918; doi:10.1038/s42004-020-00367-0)
Supplement: Supplementary file 1 — Supplementary Information [file 42004_2020_367_MOESM1_ESM.pdf]

Rapid optimisation of fragments and hits to lead compounds from screening of crude reaction mixtures

Supplementary information

Lisa M. Baker et al

Supplementary Tables

Supplementary Table 1 – The main results from the experiments. *The table shows the structures and properties of expected compounds in each of the CRMs and a summary of the results from X-ray diffraction and SPR experiments. Each row of the table is for a different CRM. CRMs 1-55 are generated from SM1 (**1**); CRMs 56-83 from SM2 (**2**). For each CRM is shown the expected structure of product (column 2), the amine reagent used (column 5), together with the heavy atom count (HAC), number of rotatable bonds (nRot), the measured yield of product in the CRM (column 6) and the predicted solubility of the product at pH5.5 and pH6.5 (columns 22 and 23). The central portion of the table shows for PDHK2 (columns 7-12) and Hsp90 (columns 14-19) the measured k_{off} by SPR and a colour coded summary of the number of SM and PR seen in the triplicate diffraction experiments, as detected by either manual or PanDDA interpretation of the electron density and the consensus classification based on the PanDDA processing used in the analyses presented in this paper; RSCC is the real space correlation coefficient for the fit of the ligand to the 2Fo-Fc map generated in the PanDDA process for a dataset from the triplicate (best PR or SM); RSCC is given a value of 0 where PanDDa did not report a fit due to low occupancy of the ligand. A magenta background is for 0, yellow for 1, orange for 2 and turquoise for 3 occurrences of either SM or PR for each CRM. A k_{off} value of 1 sec^{-1} was given to all samples with k_{off} values greater or equal to 1 sec^{-1} as this is the limit of assay sensitivity. ND (Not Determined) was for samples for which k_{off} evaluation was problematic or not possible. * the 13 CRMs (and associated purified products) used to establish crystal soaking protocols for PDHK2. # the 6 CRMs (and associated purified products) used to validate the soaking protocol for Hsp90.*

The following is a description of the data for one selected row:

Crude reaction mixture 2 (CRM2) contains compound ***11*** *at 8% yield, generated from starting material 1 (SM1,* ***1****) coupled to a tetrahydroquinoline.* ***11*** *has 22 heavy atoms and 1 rotatable bond. When CRM2 was run over an SPR surface containing PDHK2 or Hsp90, a response equivalent to a* k_{off} *of 0.169* s^{-1} *and 0.1* s^{-1} *was measured. For diffraction data collected from the triplicate soaks of PDHK2 crystals with CRM2, manual fitting gave 1 structure where the starting material was seen to bind (SM) and 2 structures where the product,* ***11****, was bound (PR). Analysis of the diffraction data using PanDDA gave the same result (1 SM and 2 PR) with the best PR structure having a real space correlation coefficient between the compound and 2Fo-Fc electron density of 0.94. For diffraction data collected from the triplicate soaks of Hsp90 crystals with CRM2, manual fitting gave 2 SM and no PR, the same for PanDDA with the best SM having an RSCC of 0.92. Compound* ***11*** *has a predicted solubility of 0.2 mM at both pH5.5 and pH6.6. Pure compound* ***11*** *was one of the compounds used to validate the soaking protocols used for the CRMs for PDHK2.*

1	2	3	4	5	6	7	8	9	10	11	12	13	14	15	16	17	18	19	20	21	22	23
						PDHK2							Hsp90								predicted solubility (mM)	
							Manual		PanDDa				Manual			PanDDa						
CRM No	PR Chemical Structure	HAC	nRot	Amine Reagent	Yield	k_{off} s^{-1} PDHK2	No SM	No PR	No SM	No PR	Used	RSCC	k_{off} s^{-1} Hsp90	No SM	No PR	No SM	No PR	Used	RSCC	No in Paper	pH5.5	pH6.5
				**SM1**		1							1							1		
1	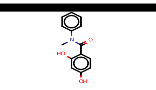	18	2	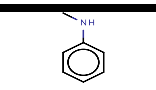	22.6	1	0	2	0	2	PR	0.97	0.08	0	2	0	2	PR	0.99		1.5	1.5
2*	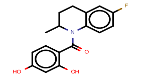	22	1	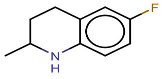	8	0.169	1	2	1	2	PR	0.94	0.1	2	0	2	0	SM	0.92	**11**	0.1	0.1
3*#	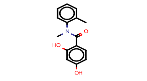	19	2	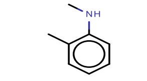	8	1	0	2	0	2	PR	0.94	0.056	1	0	0	2	PR	0.83	**12**	0.8	0.8
4	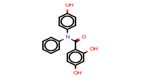	24	3	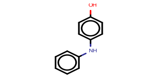	42.4	ND	2	0	0	2	PR	0	0.017	3	0	3	0	SM	0.92		0.3	0.3
5	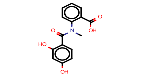	21	3	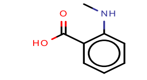	50	1	2	0	2	0	SM	0	0.04	0	0	1	0	SM	0.86		120	1000
6	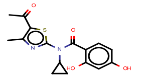	23	4	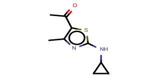	12	0.2	0	2	0	1	PR	0.94	1E-04	2	0	1	0	SM	0.93		0.2	0.2
7	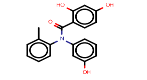	25	3	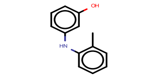	48.1	ND	2	1	1	1	PR	0.92	0.03	3	0	3	0	SM	0.89		0.1	0.1
8	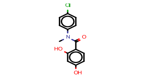	19	2	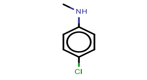	28	0.542	0	3	0	2	PR	0.93	0.09	0	3	0	3	PR	0.98		0.3	0.3
9	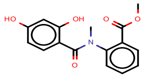	22	4	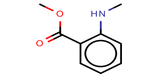	1.1	0.17	0	3	1	2	PR	0.94	0.03	1	0	0	1	PR	0.77		0.8	0.8
10	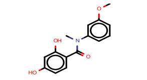	20	3	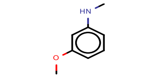	14	0.16	0	3	0	3	PR	0.96	0.067	1	0	0	2	PR	0.84		1.4	1.5
11*#	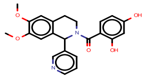	30	4	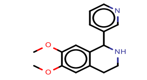	34	0.047	0	3	0	1	PR	0.97	0.23	0	0	1	0	SM	0.85	**13**	0.6	0.6
12	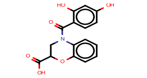	23	2	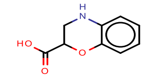	8.7	ND	3	0	3	0	SM	0.84	0.07	3	0	3	0	SM	0.91		1.0	10.0
1	2	3	4	5	6	7	8	9	10	11	12	13	14	15	16	17	18	19	20	21	22	23

1	2	3	4	5	6	7	8	9	10	11	12	13	14	15	16	17	18	19	20	21	22	23
						PDHK2							Hsp90								predicted solubility (mM)	
							Manual		PanDDa				Manual			PanDDa						
CRM No	PR Chemical Structure	HAC	nRot	Amine Reagent	Yield	k_{off} s^{-1} PDHK2	No SM	No PR	No SM	No PR	Used	RSCC	k_{off} s^{-1} Hsp90	No SM	No PR	No SM	No PR	Used	RSCC	No in Paper	pH5.5	pH6.5
			**SM1**			**1**							**1**							**1**		
13	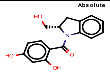	21	2	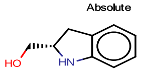	13.7	0.111	0	3	0	3	PR	0.9	1	0	2	0	2	PR	0.98		1.3	1.4
14	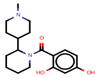	23	2	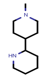	28	0.14	0	3	0	3	PR	0.94	0.012	2	0	2	0	SM	0.88		1320	390
15*	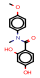	20	3	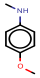	1	ND	2	0	1	1	PR	0.87	0.06	0	0	1	0	SM	0.84	**14**	1.4	1.5
16	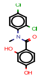	20	2	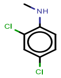	0	ND	2	0	1	0	SM	0	ND	0	0	0	0	N	0		0.1	0.1
17	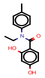	20	3	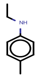	5.5	1	0	3	0	3	PR	0.96	0.03	1	2	0	3	PR	0.98		0.2	0.2
18	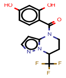	23	2	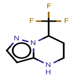	15.5	1	0	3	0	3	PR	0.96	0.06	0	3	0	3	PR	0.95		0.3	0.3
19	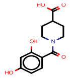	19	2	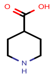	50	1	0	3	0	3	PR	0.9	0.17	2	1	2	1	PR	0.95		2377	3829
20	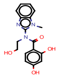	24	4	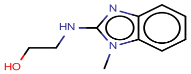	48.1	1	0	3	0	3	PR	0.94	1	3	0	3	0	SM	0.96		0.9	0.9
21	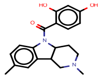	25	1	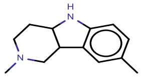	50	0.45	2	0	1	0	SM	0	0.27	1	1	0	2	PR	0.93		42.6	4.5
22	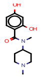	19	2	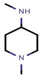	25	1	1	2	0	2	PR	0.94	0.05	1	2	0	3	PR	0.98		8353	2036
23	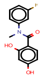	19	2	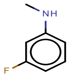	0	1	1	1	0	1	PR	0.94	0.04	0	0	0	1	PR	0.81		0.8	0.8
24	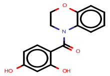	20	1	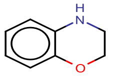	1	1	3	0	2	0	SM	0.8	0.03	1	0	2	0	SM	0.84		0.5	0.5
1	2	3	4	5	6	7	8	9	10	11	12	13	14	15	16	17	18	19	20	21	22	23

1	2	3	4	5	6	7	8	9	10	11	12	13	14	15	16	17	18	19	20	21	22	23
						PDHK2							Hsp90								predicted solubility (mM)	
							Manual		PanDDa				Manual			PanDDa						
CRM No	PR Chemical Structure	HAC	nRot	Amine Reagent	Yield	k_{off} s^{-1} PDHK2	No SM	No PR	No SM	No PR	Used	RSCC	k_{off} s^{-1} Hsp90	No SM	No PR	No SM	No PR	Used	RSCC	No in Paper	pH5.5	pH6.5
				**SM1**		1							1							1		
25	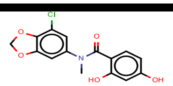	22	2	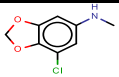	62.1	1	0	2	0	2	PR	0.89	1	0	3	0	3	PR	0.95		0.1	0.1
26*	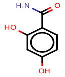	11	1	NH_3	74.4	ND	0	3	0	1	PR	0.56	1	0	0	0	0	N	0	1	166	170
27	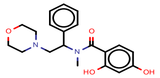	26	5	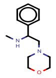	28.1	1	3	0	3	0	SM	0.91	0.6	3	0	3	0	SM	0.89		20	5.1
28	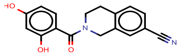	22	1	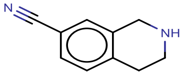	38	0.103	0	3	0	3	PR	0.96	0.4	0	3	0	3	PR	0.96		0.6	0.6
29	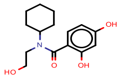	20	4	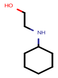	3.9	ND	2	1	1	1	PR	0.92	0.03	1	0	1	0	SM	0.89		4.5	4.6
30	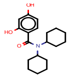	23	3	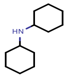	0	1	3	0	3	0	SM	0.81	0.04	1	0	1	1	PR	0.86		0.1	0.1
31	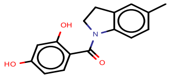	20	1	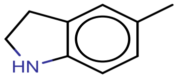	0	1	3	0	2	1	PR	0.89	ND	3	0	3	0	SM	0.89		0.2	0.2
32	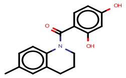	21	1	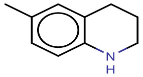	42.7	0.154	0	3	0	3	PR	0.96	0.12	0	2	0	2	PR	0.97		0.1	0.1
33	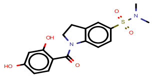	25	2	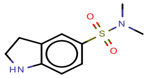	44	1	0	2	0	2	PR	0.96	0.6	0	3	0	3	PR	0.96		1.1	1.1
34	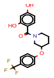	27	4	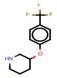	82	0.3	0	2	0	2	PR	0.95	0.03	0	3	0	3	PR	0.96		0.0	0.0
35	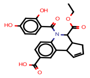	31	5	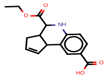	0	0.055	3	0	3	0	SM	0.86	ND	3	0	3	0	SM	0.91		1.3	13.3
36	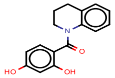	20	1	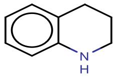	0	ND	3	0	3	0	SM	0.86	0.15	1	0	1	0	SM	0.91		0.4	0.4
1	2	3	4	5	6	7	8	9	10	11	12	13	14	15	16	17	18	19	20	21	22	23

1	2	3	4	5	6	7	8	9	10	11	12	13	14	15	16	17	18	19	20	21	22	23
						PDHK2							Hsp90								predicted solubility (mM)	
							Manual		PanDDa					Manual		PanDDa						
CRM No	PR Chemical Structure	HAC	nRot	Amine Reagent	Yield	k_{off} s^{-1} PDHK2	No SM	No PR	No SM	No PR	Used	RSCC	k_{off} s^{-1} Hsp90	No SM	No PR	No SM	No PR	Used	RSCC	No in Paper	pH5.5	pH6.5
				**SM1**		1							1							1		
37	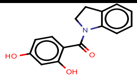	19	1	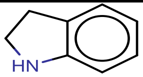	4	ND	3	0	2	1	PR	0.9	0.15	1	0	2	0	SM	0.91		0.7	0.8
38	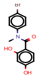	19	2	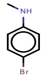	12	0.22	0	1	0	1	PR	0.88	0.06	0	0	0	1	PR	0.80		0.1	0.1
39	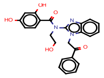	32	7	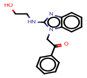	37	ND	3	0	3	0	SM	0.86	0.08	3	0	3	0	SM	0.96		0.0	0.0
40	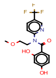	25	6	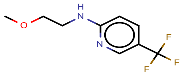	39	1	0	2	0	2	PR	0.95	0.56	0	2	0	2	PR	0.94		0.6	0.6
41	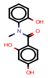	19	2	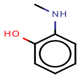	10	0.223	0	3	0	3	PR	0.97	0.02	0	3	0	3	PR	0.98		3.8	4.0
42	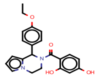	28	4	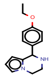	10	ND	0	2	0	2	PR	0.94	0.009	1	0	1	0	SM	0.88		0.2	0.2
43*	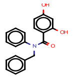	24	4	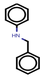	19	0.275	0	3	0	3	PR	0.96	0.09	0	3	0	3	PR	0.92	**15**	0.0	0.0
44	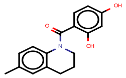	21	1	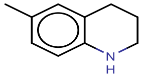	1.7	1	2	1	2	1	PR	0.93	0.1	2	0	2	0	SM	0.88		0.1	0.1
45	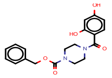	26	4	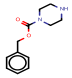	29	1	0	3	0	3	PR	0.95	0.4	0	0	0	0	N	0		0.4	0.4
46	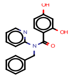	24	4	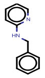	35	0.268	0	3	0	2	PR	0.96	0.5	0	3	0	3	PR	0.97		0.1	0.1
47	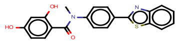	27	3	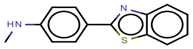	48	0.44	1	2	1	2	PR	0.93	0.28	0	2	0	3	PR	0.93		0.0	0.0
48	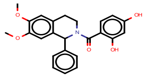	30	4	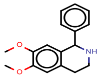	82	0.285	0	3	0	3	PR	0.97	0.04	2	0	2	0	SM	0.90		0.0	0.0
1	2	3	4	5	6	7	8	9	10	11	12	13	14	15	16	17	18	19	20	21	22	23

1	2	3	4	5	6	7	8	9	10	11	12	13	14	15	16	17	18	19	20	21	22	23
						**PDHK2**							**Hsp90**								predicted solubility (mM)	
							**Manual**		**PanDDa**				**Manual**			**PanDDa**						
**CRM No**	**PR Chemical Structure**	**HAC**	**nRot**	**Amine Reagent**	**Yield**	**k_{off} s^{-1} PDHK2**	**No SM**	**No PR**	**No SM**	**No PR**	**Used**	**RSCC**	**k_{off} s^{-1} Hsp90**	**No SM**	**No PR**	**No SM**	**No PR**	**Used**	**RSCC**	**No in Paper**	pH5.5	pH6.5
			**SM1**			**1**							**1**							**1**		
49	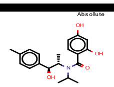	25	5	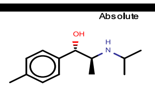	15	ND	3	0	2	1	PR	0.92	0.03	3	0	3	0	SM	0.94		0.2	0.2
50	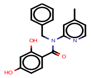	25	4	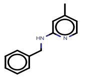	30	0.075	0	2	0	1	PR	0.96	0.06	0	0	1	0	SM	0.84		0.0	0.0
51	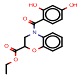	25	4	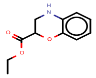	0	1	3	0	3	0	SM	0.87	0.01	0	0	0	0	N	0		0.1	0.1
52	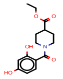	21	4	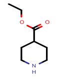	7	1	0	3	0	2	PR	0.94	0.08	1	1	1	2	PR	0.93		11.7	12.0
53	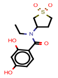	20	3	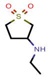	7	0.08	0	3	0	3	PR	0.92	0.2	0	3	0	3	PR	0.94		20.6	21.1
54	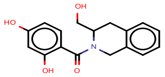	22	2	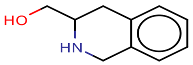	100	1	0	3	0	3	PR	0.95	0.03	0	2	0	3	PR	0.95		1.9	2.0
55	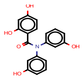	25	3	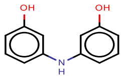	36.1	0.144	1	1	1	0	SM	0.8	ND	2	0	2	0	SM	0.89	**25**	0.7	0.7

1	2	3	4	5	6	7	8	9	10	11	12	13	14	15	16	17	18	19	20	21	22	23

1	2	3	4	5	6	7	8	9	10	11	12	13	14	15	16	17	18	19	20	21	22	23
						PDHK2							Hsp90								predicted solubility (mM)	
							Manual		PanDDa					Manual		PanDDa						
CRM No	PR Chemical Structure	HAC	nRot	Amine Reagent	Yield	k_{off} s^{-1} PDHK2	No SM	No PR	No SM	No PR	Used	RSCC	k_{off} s^{-1} Hsp90	No SM	No PR	No SM	No PR	Used	RSCC	No in Paper	pH5.5	pH6.5
				**SM2**		**0.083**							**0.2**							**2**		
**56*#**	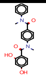	28	4	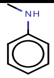	84	ND	0	2	0	2	PR	0.95	0.06	0	3	0	3	PR	0.96	**16**	0.0	0.0
57	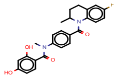	32	3	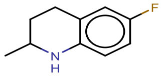	39	0.189	3	0	3	0	SM	0.96	0.03	3	0	3	0	SM	0.97	**26**	0.0	0.0
58*	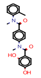	29	4	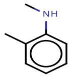	100	0.066	2	1	2	1	PR	0.96	0.1	3	0	2	0	SM	0.98	**17**	0.0	0.0
59	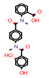	31	5	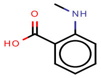	0	0.24	2	1	3	0	SM	0.96	0.16	2	0	2	0	SM	0.96		1.3	13.9
60*#	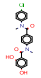	29	4	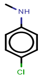	100	0.15	0	3	1	2	PR	0.94	0.16	1	2	0	3	PR	0.94	**18**	0.0	0.0
61	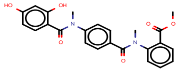	32	6	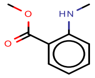	27	0.185	2	0	2	0	SM	0.96	0.1	3	0	3	0	SM	0.98		0.0	0.0
62	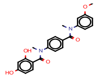	30	5	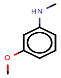	76.9	0.186	3	0	3	0	SM	0.96	0.2	3	0	3	0	SM	0.97		0.0	0.0
63*#	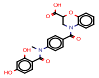	33	4	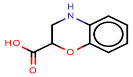	8.7	0.119	1	2	0	3	PR	0.95	0.012	2	0	2	0	SM	0.98	**19**	1.0	10.0
64	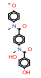	30	5	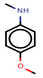	9	0.734	3	0	3	0	SM	0.96	0.19	3	0	3	0	SM	0.98		0.0	0.0
65	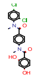	30	4	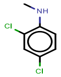	0	0.646	3	0	3	0	SM	0.96	0.26	3	0	3	0	SM	0.98		0.0	0.0
66	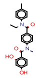	30	5	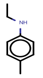	100	0.058	1	0	1	0	SM	0.95	0.19	0	3	0	3	PR	0.96		0.0	0.0
67	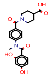	29	4	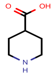	71.6	0.089	1	2	2	1	PR	0.94	0.09	3	0	3	0	SM	0.97		14	140
1	2	3	4	5	6	7	8	9	10	11	12	13	14	15	16	17	18	19	20	21	22	23

1	2	3	4	5	6	7	8	9	10	11	12	13	14	15	16	17	18	19	20	21	22	23
						PDHK2							Hsp90								predicted solubility (mM)	
							Manual		PanDDa				Manual			PanDDa						
CRM No	PR Chemical Structure	HAC	nRot	Amine Reagent	Yield	k_{off} s^{-1} PDHK2	No SM	No PR	No SM	No PR	Used	RSCC	k_{off} s^{-1} Hsp90	No SM	No PR	No SM	No PR	Used	RSCC	No in Paper	pH5.5	pH6.5
				**SM2**		**0.083**							**0.2**							**2**		
68*	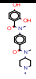	29	4	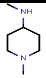	8.8	0.143	3	0	3	0	SM	0.96	0.12	3	0	3	0	SM	0.97	**20**	152	16
69	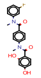	29	4	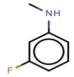	90	0.199	0	3	0	3	PR	0.95	0.04	0	3	0	3	PR	0.95		0.0	0.0
70	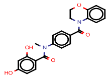	30	3	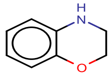	88	0.688	0	3	0	3	PR	0.95	0.015	1	2	0	3	PR	0.89		0.0	0.0
71	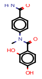	21	3	NH_3	78	0.083	0	3	0	3	PR	0.96	0.3	0	2	0	2	PR	0.96	**2**	1.0	1.0
72*	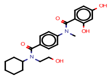	30	6	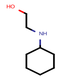	8.3	0.182	2	1	3	0	SM	0.96	0.12	3	0	3	0	SM	0.98	**21**	0.0	0.1
73	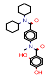	33	5	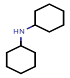	1	0.168	3	0	3	0	SM	0.97	0.3	3	0	3	0	SM	0.97		0.0	0.0
74	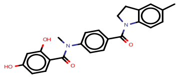	30	3	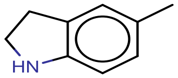	0	0.28	2	0	2	0	SM	0.96	0.26	2	0	2	0	SM	0.97		0.0	0.0
75	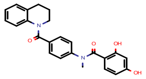	30	3	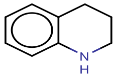	3.3	0.158	3	0	3	0	SM	0.97	0.013	3	0	3	0	SM	0.96	**27**	0.0	0.0
76*	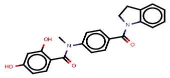	29	3	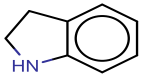	43	0.165	1	2	1	1	PR	0.93	0.018	3	0	2	1	PR	0.97	**22**	0.0	0.0
77	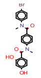	29	4	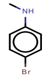	53	0.46	0	3	0	3	PR	0.95	0.2	0	3	0	3	PR	0.90		0.0	0.0
78	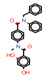	34	6	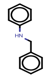	88	0.202	3	0	3	0	SM	0.97	0.07	1	2	1	2	PR	0.93		0.0	0.0
79	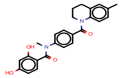	31	3	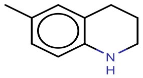	30	0.447	3	0	3	0	SM	0.95	0.08	3	0	3	0	SM	0.97		0.0	0.0
1	2	3	4	5	6	7	8	9	10	11	12	13	14	15	16	17	18	19	20	21	22	23

1	2	3	4	5	6	7	8	9	10	11	12	13	14	15	16	17	18	19	20	21	22	23
						PDHK2							Hsp90								predicted solubility (mM)	
							Manual		PanDDa				Manual			PanDDa						
CRM No	PR Chemical Structure	HAC	nRot	Amine Reagent	Yield	k_{off} s^{-1} PDHK2	No SM	No PR	No SM	No PR	Used	RSCC	k_{off} s^{-1} Hsp90	No SM	No PR	No SM	No PR	Used	RSCC	No in Paper	pH5.5	pH6.5
			**SM2**			**0.083**							**0.2**							**2**		
80	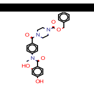	36	6	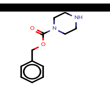	37	0.548	2	1	2	1	PR	0.96	0.04	2	0	2	0	SM	0.97		0.0	0.0
81	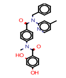	35	6	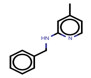	10	0.14	3	0	2	0	SM	0.96	0.2	3	0	3	0	SM	0.96		0.0	0.0
82	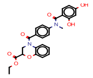	35	6	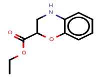	27	0.058	1	2	0	1	PR	0.94	0.004	3	0	3	0	SM	0.97		0.0	0.0
83	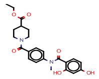	31	6	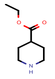	8	0.074	3	0	3	0	SM	0.96	0.05	3	0	3	0	SM	0.97	**28**	0.1	0.1

Supplementary Table 2 - Data collection and refinement statistics for structures in Figures 1 and 5

Rfree is the R factor calculated using 5% of the reflection data chosen randomly and omitted from the refinement process. Rms bond lengths and angles are the deviations from ideal values. Values in parentheses are for the highest resolution bin.

**Complex**	**Hsp90 + 1**	**Hsp90 + 2**	**PDHK2 + 1**	**PDHK2 + 2**	**PDHK2 + 23**	**PDHK2 + 24**	**PDHK2 + CRM 11**
PDB code	6TN4	6TN5	6TN0	6TN2	6TMP	6TMQ	6TMZ
**Data Collection**							
Space Group	I 2 2 2	I 2 2 2	P 64	P 64	P 64	P 64	P 64
Unit Cell dimensions (a,b,c)(Å)	65.693 89.422 99.829	65.720 89.530 100.025	109.051 109.051 83.945	108.516 108.516 83.676	109.292 109.292 83.654	110.368 110.368 83.863	110.379 110.379 83.679
Unit Cell dimensions (α,β,γ)(°)	90.0 90.0 90.0	90.0 90.0 90.0	90.0 90.0 120.0	90.0 90.0 120.0	90.0 90.0 120.0	90.0 90.0 120.0	90.0 90.0 120.0
Resolution (Å)	1.274	1.165	1.905	1.768	2.076	2.112	2.710
Rmerge	0.042 (0.748)	0.057 (0.654)	0.056 (1.241)	0.067 (1.065)	0.092 (1.197)	0.127 (1.857)	0.105 (1.333)
I/σ(I)	17.6 (1.6)	14.0 (1.6)	20.4 (1.8)	17.2 (2.0)	16.8 (1.9)	13.8 (1.5)	17.1 (1.7)
Completeness	95.9 (68.3)	91.1 (60.9)	94.3 (71.5)	92.9 (63.8)	93.9 (76.2)	83.3 (69.9)	91.8 (83.1)
Redundancy	6.3 (5.4)	6.1 (3.7)	10.3 (10.5)	10.4 (9.5)	10.2 (9.2)	10.1 (9.2)	10.0 (10.2)
**Refinement**							
Resolution (Å)	1.274	1.165	1.905	1.768	2.076	2.112	2.710
No. reflections all/free	57101 / 3067	74921 / 3900	26469 / 1332	29137 / 1530	19050 / 934	18329 / 889	11031 / 556
R-factor/R-free	0.171 / 0.198	0.169 / 0.184	0.176 / 0.221	0.171 / 0.211	0.171 / 0.240	0.176 / 0.234	0.192 / 0.258
No Atoms Protein	3343	3419	5433	5495	5563	5478	5414
No Atoms Ligand/Ions	19	36	79	96	103	112	103
No Atoms water	370	395	146	204	140	150	31
Protein B-factor	20.5	15.91	50.44	44.11	51.11	49.28	71.1
Ligand B-factor	24.62	11.5	59.67	46.76	49.3	52.77	59.62
Water B-factor	35.42	31.58	49.77	45.70	45.32	45.62	46.24
**RMS Deviations**							
Bonds (Å)	0.0151	0.0179	0.0089	0.0093	0.0142	0.0153	0.0076
Angles (°)	1.964	2.159	1.538	1.525	1.8900	1.9712	1.602

Supplementary Figures

Supplementary Figure 1 - Structures of compounds explicitly referenced in the manuscript and not shown elsewhere

11

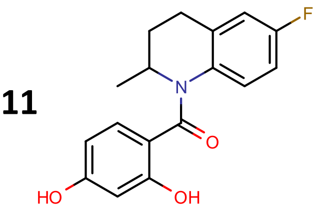

12

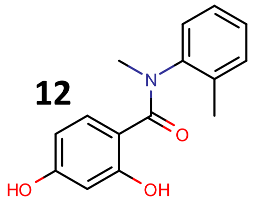

13

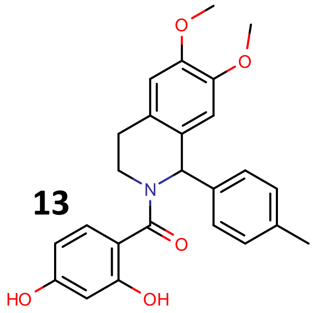

14

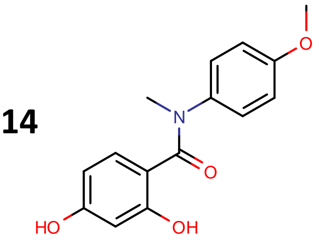

15

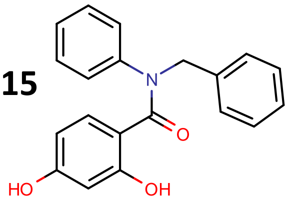

16

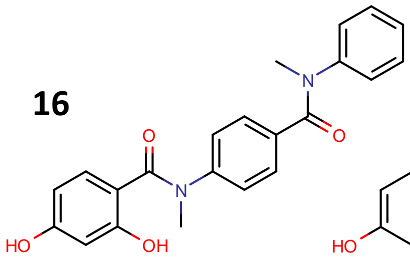

17

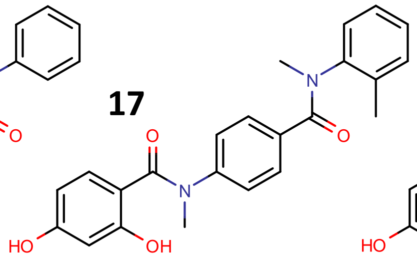

18

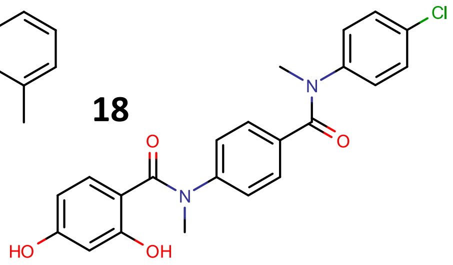

19

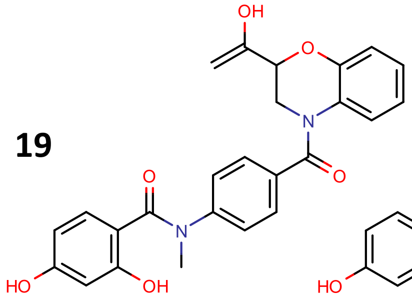

20

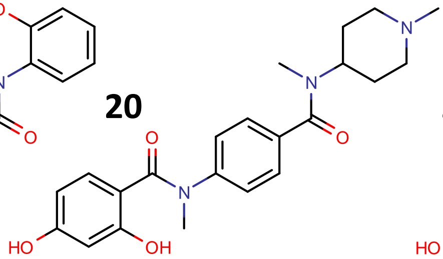

21

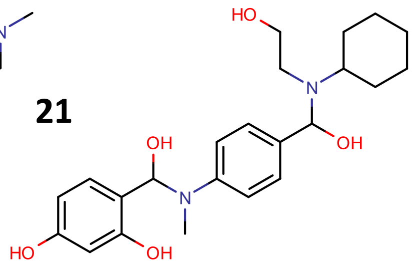

22

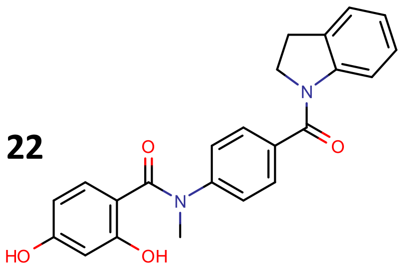

25

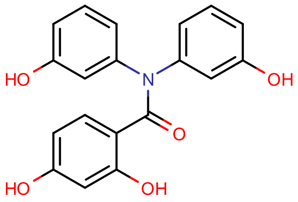

26

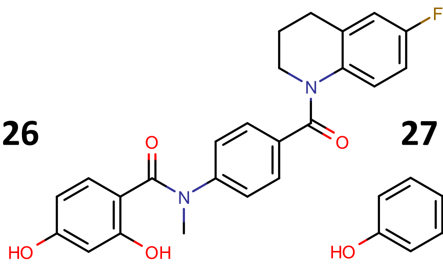

27

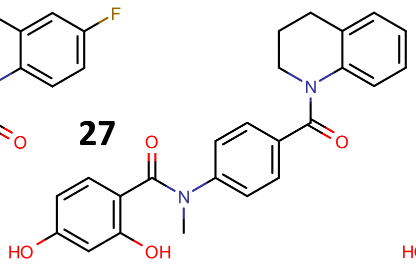

28

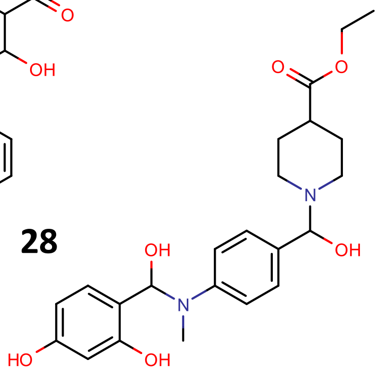

Supplementary Figure 2 - Soak time has little influence on resolution of data collected from the soaked crystals

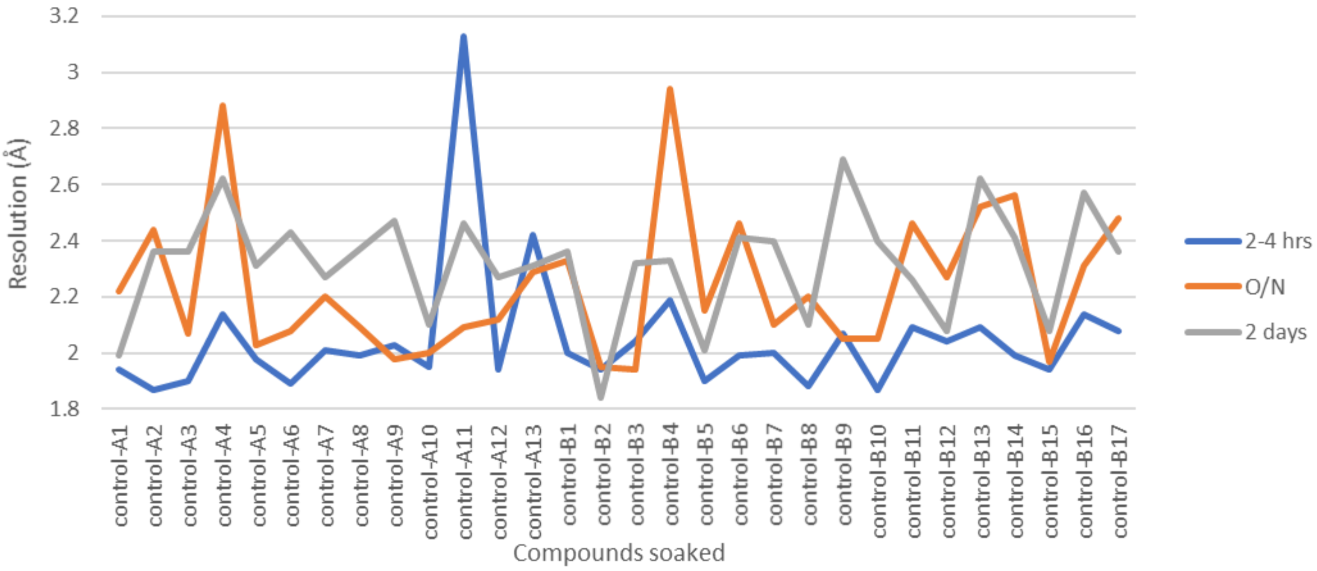

Legend to Supplementary Figure 2: Plot of the best resolution of diffraction data collected for each control compound vs soak time for the control samples for PDHK2 at 2mM concentration. Compounds A1 to A13 are CRM and B1 to B17 purified compounds.

Supplementary Figure 3 - A crystal soaked for 2 days generates clearer electron density for the ligand than a 2-4 hour soak.

(a)(i) PDHK2 – (2-4hours) :

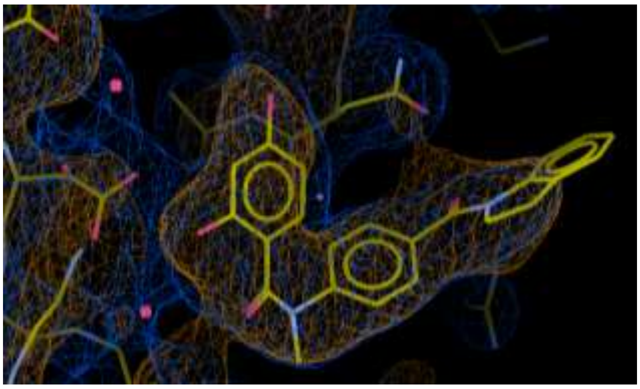

(ii) PDHK2 – (2 days) : PanDDA

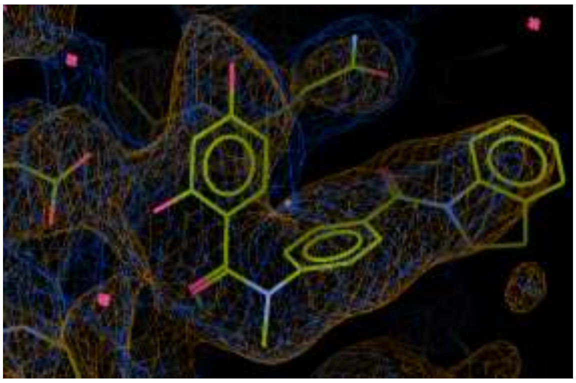

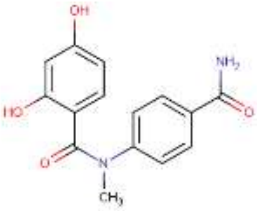

SM2 (**2**)

CRM 76

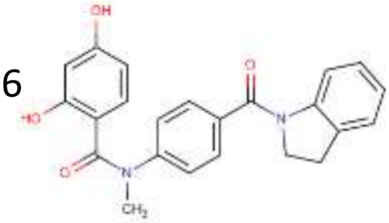

(b) (i) PDHK2 (2-4hours) :

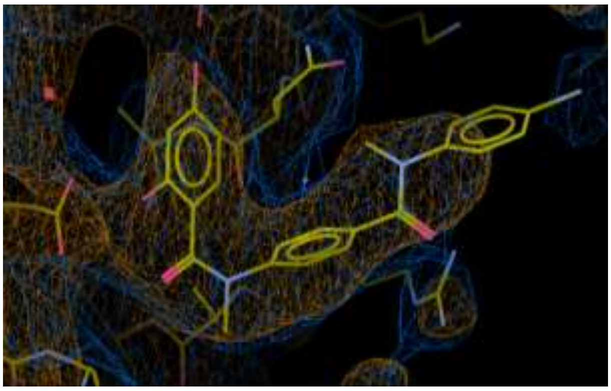

(ii) PDHK2 (2 days) : PanDDA

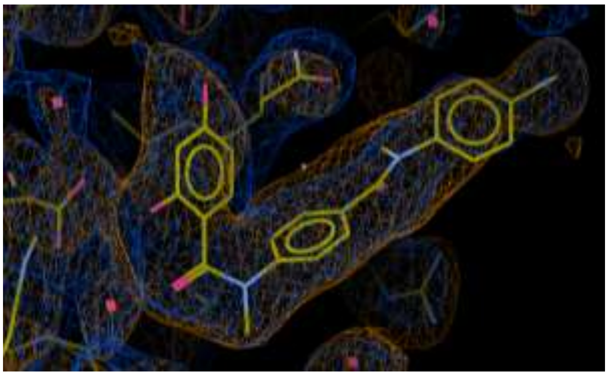

19 – pure of CRM

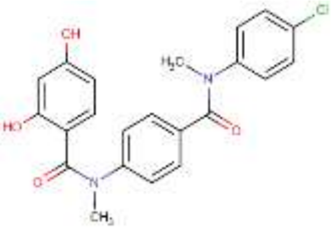

Legend to Supplementary Figure 3: A comparison of the PanDDA electron density maps observed for (a) CRM 76 and (b) pure compound ***19*** *(the equivalent of the product from CRM 60) soaked in PDHK2 at 2mM for (i) 2-4hrs vs (ii) 2-days. The structures are drawn with yellow carbon atoms, red oxygen atoms and blue nitrogen atoms. The 2Fo-Fc electron density contoured at 1.4σ is shown in blue, the PanDDA event map contoured at 2.0σ is shown in orange.*

Supplementary Figure 4 - Manual fitting sometimes correctly identifies a product binding where the automated process does not.

(i) PDHK2 – Manual

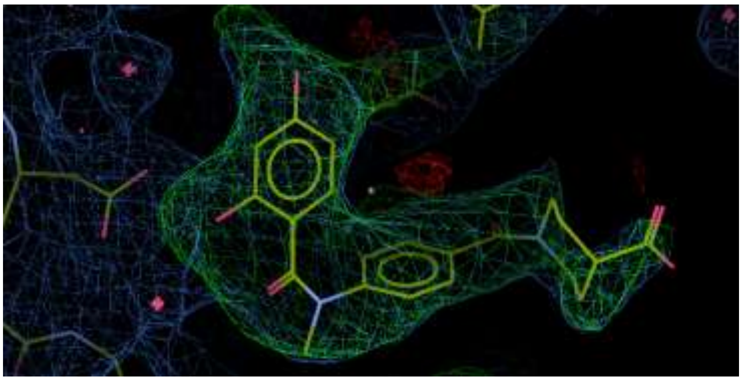

2fofc (blue) = 1.4σ, fofc (green) = 2.5σ

(ii) PDHK2 – PanDDA

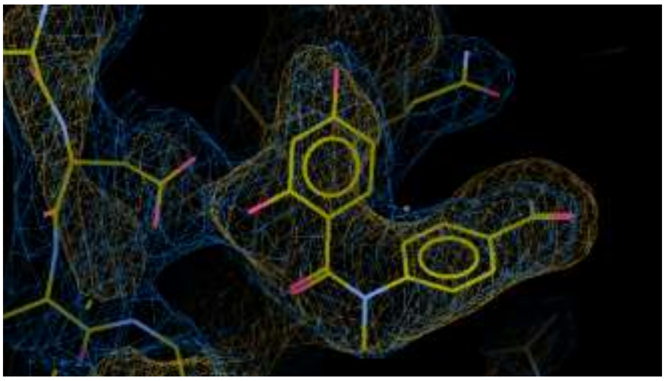

2fofc (blue) = 1.4σ,
PanDDA map (orange) = 2.0σ

CRM 67

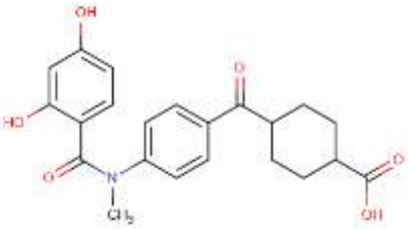

SM2

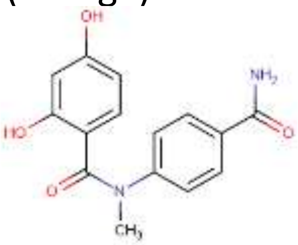

Legend to Supplementary Figure 4: A comparison of the electron density for CRM 67 soaked in PDHK2 for (i) manually generated maps vs (ii) PanDDA average maps. In this example, the XCE pipeline chose a lower resolution data set (2.7Å) from the autoprocessing results compared to that chosen for the manual study (2.5 Å). This resulted in fitting of starting material in the PanDDA maps, compared to the manual map where the product of CRM 67 could be fitted. The structures are drawn with yellow carbon atoms, red oxygen atoms and blue nitrogen atoms. The 2Fo-Fc electron density contoured at 1.4σ is shown in blue, Fo-Fc electron density contoured at 2.5σ is shown in green and the PanDDA event map contoured at 2.0σ is shown in orange.

Supplementary Figure 5 (a) – PR or SM is not related to 1/k_{off} for the CRM

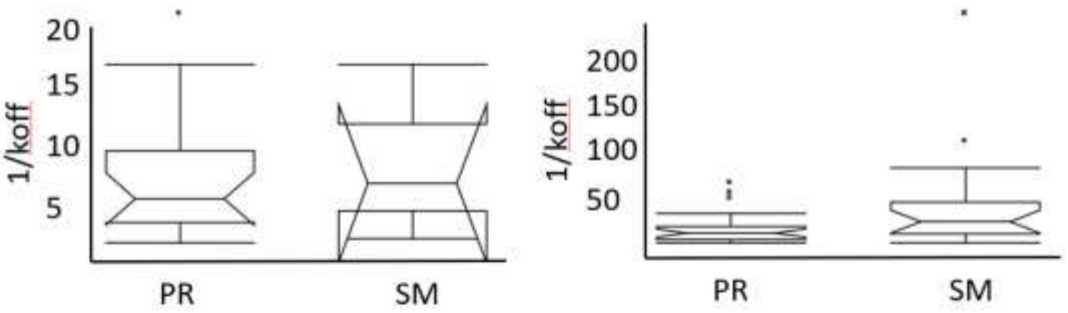

Legend to Supplementary Figure 5(a) – Notched boxplots for the distributions for SM and PR, narrowing the box around the median, showing the first and third quartile (Left - PDHK2, Right - Hsp90). Notches extend beyond the box limits due to small sample size for PDHK (Left). The number of SM for PDHK2 precludes a secure statistical calculation for PDHK2. For Hsp90, a 2-sample Kolmagorov-Smirnoff test (see Methods) fails to reject the null hypothesis (that PR and SM are from the same distribution of 1/k_{off}) at the 95% confidence level ($D_{PR,SM}$ = 0.32, p=0.205).

Supplementary Figure 5 (b) – The dependence of PR or SM on HAC or nRot in the product compound

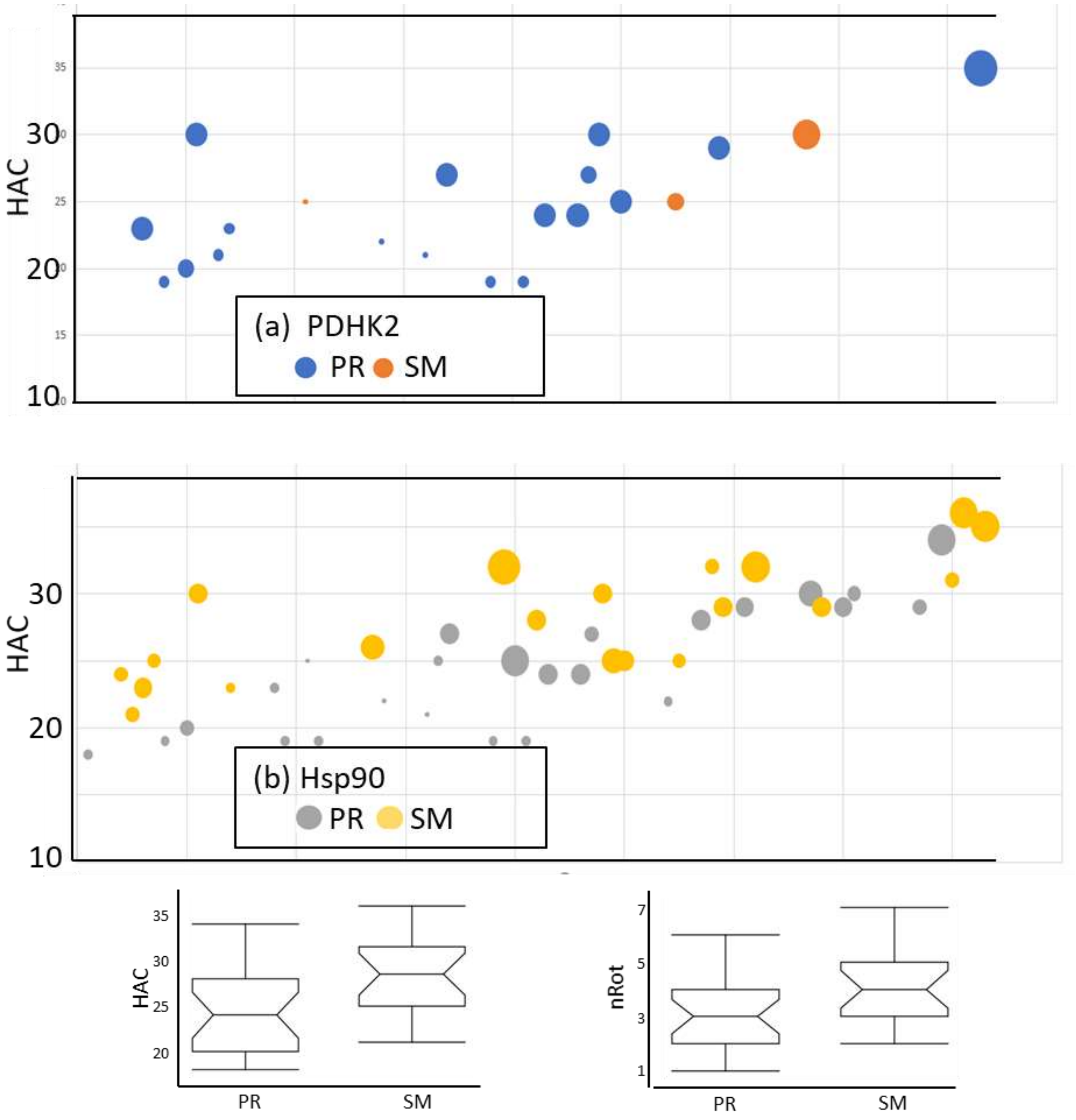

Legend for Supplementay Figure 5(b) – Plot of HAC for those CRMs that gave a slower k_{off} product than starting material and a yield > 10% for each of (a) PDHK2 (blue PR) and (b) Hsp90 (grey PR) with size of marker scaled by nRot (between 1-6) for SM and PR. The number of SM satisfying the k_{off} and yield criteria for PDHK2 precludes a secure statistical calculation. The notched boxplots are for Hsp90 of the distributions for SM and PR, narrowing the box around the median, showing the first and third quartile. The HAC was 24.4 +/- 4.4 and nRot 3.0 +/- 1.4 for CRMs giving PR and the HAC was 28.1 +/- 4.1 and nRot 4.1 +/- 1.3 for CRMs giving SM (mean +/- standard deviation).A 2-sample Kolmagorov-Smirnoff test (see Methods) fails to reject the null hypothesis (that PR and SM are from the same distribution) at the 95% confidence level for HAC ($D_{PR,SM}$ = 0.39, p=0.068), but does reject the null hypothesis for nRot ($D_{PR,SM}$ = 0.43, p=0.033).

Supplementary Figure 6 - Omit maps for the structures in Figures 1 and 5 of the manuscript

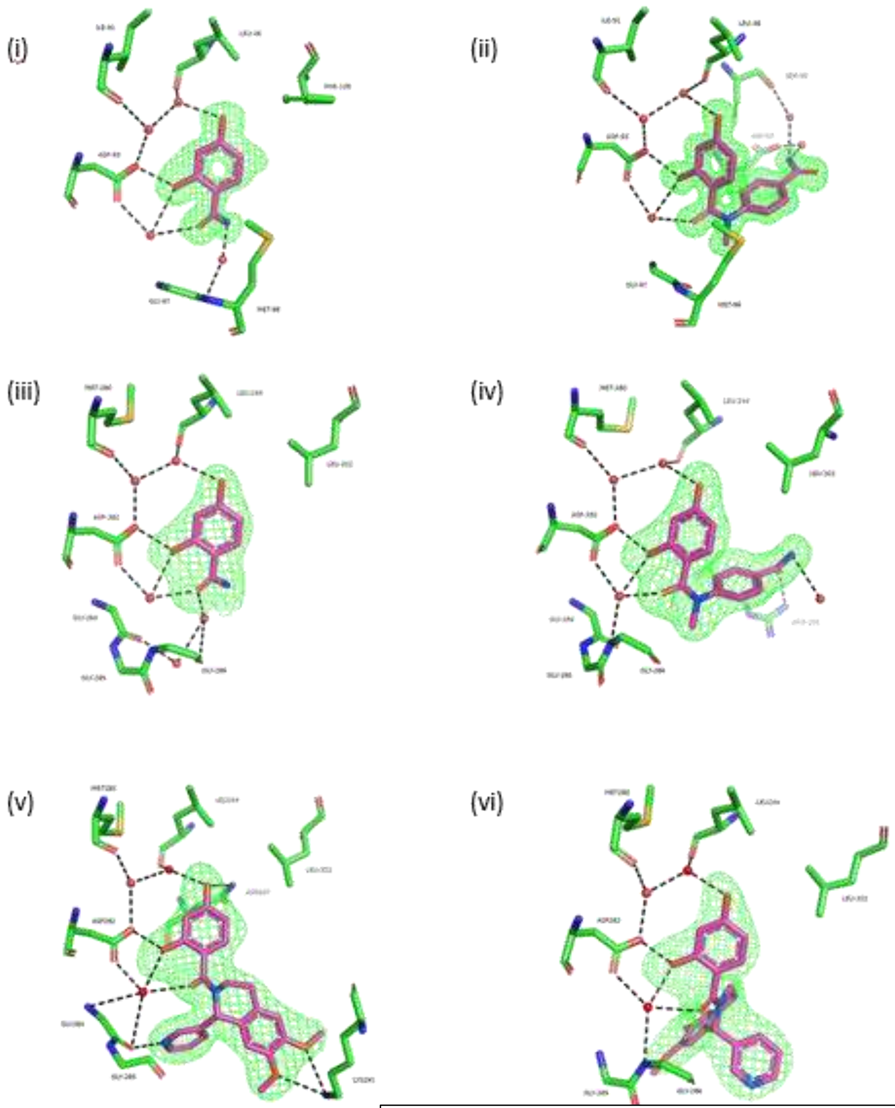

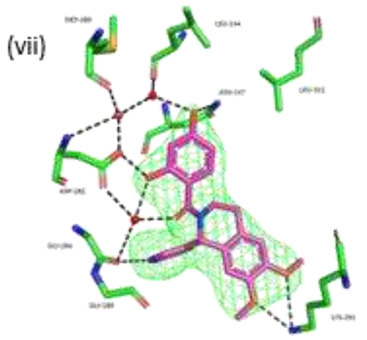

Legend for Supplementary Figure 6 Omit maps for the structures shown in Figure 1 and Figure 5 of the manuscript. Shown are compounds (magenta carbon atoms) and water molecules and amino acids from the proteins which are within 3.5Å of the compound (with green carbon atoms) and with red oxygen, blue nitrogen and yellow sulphur atoms; F_{obs}-F_{calc} electron density is contoured at 3σ for the ligand in light green; hydrogen bonding between ligand, protein and solvent is shown with black dashed lines. (i) and (ii) SM1 and SM2 bound to Hsp90; (iii) and (iv) SM1 and SM2 bound to PDHK2; (v) pure eutomer ***23****, (vi) pure distomer* ***24*** *and (vii) CRM number 11 bound to PDHK2. See main manuscript for PDB codes and resolution.*

Supplementary Notes

Supplementary Note 1: Rationale for performing all crystal soaking experiments in triplicate.

We have previously conducted structure-guided medicinal chemistry projects to optimise hits discovered from screening a fragment library against both Hsp90 and PDHK2 using ligand-observed NMR spectroscopy[1, 2]. The optimisation process was supported by determining structures of protein-ligand complexes from both crystal soaking and co-crystallisation. Obtaining structures by ligand soaking is dependent on many different factors, including ligand solubility, buffers used, the nature of the binding site, the constraints of the crystal system, requirements for conformational change to accommodate the ligand and the time and concentration of the soak. These factors variously affect the thermodynamics but also the kinetics of binding. We sometimes observe that multiple ligand soaks can be required to determine a protein-ligand complex structure for a ligand that is shown to bind by other techniques. We therefore reviewed the historical data on soaking experiments for these two targets within Vernalis to suggest protocols for the XChem experiments but also to characterise the success rate for obtaining protein-ligand structures. These experiments were not conducted systematically (or comprehensively) but the same crystal forms and similar soaking conditions were used within each project[2, 3], with structures determined and refined as published.

For Hsp90, structure determination by soaking of apo-crystals had previously been attempted for 328 different compounds shown to bind to Hsp90 by NMR spectroscopy or fluorescence polarisation assay. Protein-ligand crystal structures were obtained for 163 compounds on the first attempt, 16 on the second and 6 on the third attempt. A structure was not obtained for the remaining 143 compounds. This shows that if only one soak had

been attempted, a structure would have been obtained for 50% of the compounds with a few more structures obtained from multiple experiments. For PDHK2, 481 different compounds were soaked into apo-crystals and 271 protein ligand structures were obtained on the first and 7 on the second attempt. A structure was not obtained for the remaining 203 compounds.

We have not considered variability in compound solubility in assessing this historical data. However, the success rates suggest that for these crystal systems, a protein-ligand crystal structure is usually obtained after just one experiment, if a structure is to be obtained at all. We decided, however, to perform the XChem soaking experiments in triplicate to allow for some experimental error such as poor crystal quality, mishandling of crystals in the robotics or mechanical failure to collect a dataset.

Supplementary Note 2 - Calculation of the efficiency savings from the CRM Approach

The CRM approach results in efficiency improvements, both in terms of materials (and associated waste disposal), and time. Additionally, the highly parallel nature of the approach allows for cheminformatics to streamline the process and increase efficiency.

The following description and calculation justifies the statement in the manuscript that *"the reactions reported here for 83 CRMs took ~5 FTE days to set up and analyse, compared to an estimate of 15-20 FTE days for preparation, purification, characterisation and preparation of separate stock solution for each of the samples, additionally saving 35-50 litres of various solvents for work-up and purification, and ~7-fold saving of reagents and solvents for synthetic operations.*"

We describe each step of the process below, and quantify where possible the time and materials savings afforded by using the process. The calculations are by nature somewhat subjective, and approximate. We have tried to detail the assumptions and approximations used throughout.

It is also worth considering that without this process, fewer compounds are likely to be actually made and tested, particularly as we have demonstrated here and in previous manuscripts[4, 5] that we can sometimes detect meaningful improvements in compound binding when the product was not detected by LCMS, meaning that in all likelihood the compound would not have been tested under a traditional protocol. In addition to the quantifiable costs of materials and time, there is also a potential cost in terms of missed information, and potentially ultimately project progression, which cannot be quantified.

Library Design

We have immediate access to around 15,000 building blocks in our chemical stores. Commercial vendors also offer aggregation and reformatting services, with turnaround times varying from days to several weeks, providing access to millions of compounds. Additionally, due to the small amount of reagent required, compounds listed in vendor catalogues as screening samples can also be used as building blocks, potentially added diversity to the library. The CRM approach means that more compounds can be screened

rapidly in the early stages of a project, when understanding of structure-activity relationships (SAR) is potentially low. Therefore, enumeration of large libraries, filtering by ΔHAC and other desired properties (e.g. N+O count, cLogP/cLogD) followed by a fingerprint-based diversity selection allows for relatively rapid library design.

We have implemented an interactive KNIME workflow deployed *via* our KNIME server webportal, which allows the user to enter a core starting material and select a reagent source, and then choose from the available reaction types (decided based on substructure matches in entered core and reagent source), and deprotections, based on protecting groups found in the enumerated library. The products are then filtered to remove compounds known to our own internal or public databases. The user is then presented with a set of structural alerts from ChEMBL, for which matches are found in the enumerated, deprotected library, and can select any for which matches are to be removed from the library. With the pre-filtered library now in place, the user can select the tolerated values of ΔHAC and other desired properties. Finally, the user chooses the fingerprint type to use, and the number of compounds required in the final library, along with any additional required parameters for the settings (e.g. conformer generation options, should a fingerprint require 3D conformers).

A summary report showing ΔHAC, ΔN+O count and cLogP profile is generated, along with the fingerprint bit coverage for the whole library and diverse subset. All discarded compounds are reported in a JChem for Excel workbook, along with reagent pick lists, and sd-files for direct use in the library synthesis stage of the E-Workbook experiment.

It is difficult to quantify the efficiency saving of this route, however manually “eyeballing” a list of potentially 5000 enumerated library products to select a subset which might be of interest is certainly a time-consuming process. Alternatively, a docking campaign and analysis of results may take a number of days, and at an early stage in a project is likely to be based on limited structural information availability. This process can take as little as 15 minutes using the KNIME workflow.

Reagent picking and plating

If a set of library reagents is ordered in a 96-well format, the time spent on this step is the time awaiting delivery, when project resource can be deployed on other tasks, e.g. library condition optimisation.

For in-house reagent sets, the KNIME workflow above generates a list of reagents sorted by storage shelf location. It is possible to weigh the 1 mL matrix tubes for a typical 96-member library, locate and aliquot out a sample of the reagent, reweigh and dissolve within 1 working day. (Dispensing an accurate amount is not required, simple aliquoting out a small amount of reagent and reweighing is sufficient)

Synthesis

With a plated set of reagents pre-dissolved in an appropriate solvent, multi-way pipetting makes the time to dispense reagents negligible. A set of reactions with 1 common core, a plated reagent set and 2 reagents to be added sequentially to all wells can be pipetted manually in < 10 minutes. Again, a direct comparison with "traditional" approaches is difficult to quantify, but it is almost certain that the larger volumes will preclude the use of multiway pipettes, and longer times will be required.

We will assume a typical reaction scenario based on 5 µmol scale for CRM (we used 10 µM in this paper to ensure adequate material supply for method development across multiple biophysical techniques, however we routinely use 5 µmol scale, 2.5 µmol or even 1 µmol depending on the availability of material and reliability of the chemistry), and compare with a traditional approach targeting 5 mg submission of pure material for each sample, and base this on an average recovery of 50%. Based on the library of SM1 in this paper, the average MWt of product is ~312 Da, corresponding to ~ 32 µmol / reaction, or ~6.5-fold saving of reagents and solvents. Assuming that solvent evaporation speeds are approximately linear with solvent volume, the same ~ 6.5-fold material saving will translate into a significant time saving. For 2.5 µmol scale CRM, the saving is thus ~13-fold, and for 1 µmol scale, even greater at ~ 32.5-fold.

Additionally, whilst the workup for this procedure is unchanged, for many procedures an aqueous workup step will be required prior to purification, adding additional time, reagent and solvent costs.

This analysis does not account for the increased amount of materials required for the synthesis of a common core starting material. In the CRMs presented here, the syntheses were relatively simple, comprising 1 or 4 high-yielding steps with straightforward purification. However, templates may require more complex syntheses (more steps, poorer yields, expensive, toxic or unstable reagents, difficult purifications) in order to position an appropriate synthetic handle into a fragment hit. When this is the case, the benefit of being able to make a large number of compounds with a small amount of 'expensive' core becomes more pertinent (the above fold savings translate to fold increase in the number of compounds for a given quantity of core starting material – 10 mg might be sufficient for 1 pure compound, or as many as 30 CRMs)

Purification

For CRM, this step takes no time or materials

For a traditional approach, we consider 2 scenarios. In the first, the chemist makes compounds in small batches, and purifies ~67% of them by flash chromatography. The remaining samples require prep HPLC purification.

We assume flash chromatography uses 4g pre-packed Silica cartridges. A Biotage Combiflash Rf system standard run for such a column requires a 15.7 min run at 18 mL/min solvent flow rate, and an equilibration volume of 33.6 mL. 64 such samples equates to ~20.3 L of solvent (not including solvent used for sample loading and LCMS analysis of fractions to locate product), and will take at least 3 working days to complete.

The remaining 32 samples may require 2 or more injections on a preparative scale HPLC system, under the conditions described above in this SI. Each injection runs a 10 minute gradient at a flow rate of 20 ml/min. Injections are separated by a wash step of around 2 minutes, equating to 12 minutes and 240 mL solvent for each injection. For 32 samples at two injections each, this corresponds to a further 15.4 L of solvent, and assuming the instrument can be kept running continuously, 12.8 hours of purification. Further time will be required to locate, combine and evaporate individual fractions as above.

In the second approach, all 96 are submitted directly for HPLC purification. Using the same assumptions as above, this equates to 46 L of solvent, and ~38.4 hours of instrument

time. Again, further time will be required to locate, combine and evaporate individual fractions as above.

In the non-CRM approach, there are also manual handling losses as the reaction mixture is transferred to workup, evaporated, loaded to purification instrument, fractions combined, re-evaporated and transferred to submission vials and dried thoroughly, whereas in the CRM approach, the entire synthesis process occurs in the submission vial.

QC & sample submission

For the CRM approach, QC comprises a single LCMS analysis as described above. Each sample is separated by an ~1.5 minute gap while the instrument re-equilibrates and loads the following sample, on top of the 2 minute runtime. Allowing 1 mL / min flowrate between samples, 1.3 mL / min for 1.4 mins of the data acquisition phase, and 1.6 mL / min for the remaining 0.6 min gives a total solvent use for each sample of ~4.3 mL, i.e. a total volume of ~ 400 mL. This presents no saving compared with an LCMS sample for each pure compound. However, analysis time is greatly reduced in the CRM case (see below)

Each pure compound will be subjected to NMR analysis, using ~0.6 mL of deuterated solvent per sample, a glass NMR tube and around 5 minutes instrument time (including processing and sample change, shimming etc). This amounts to ~60 mL of solvent and 8 hours of instrument time, with subsequent analysis time to determine the correct compound, purity and nature and quantity of any residual solvent.

Finally, each pure compound must have an aliquot weighed accurately, and dissolved in the submission solvent. This is a time-consuming process, which we estimate could take around 5 minutes per sample to complete.

Informatics & Electronic Laboratory Notebook (ELN) Writeup

In our initial CRM studies, the entire process was conducted manually. Each reagent and product were drawn into the EWB, and each LCMS sample was manually assessed for purity, the results of which could be subjective. The analysis of the QC data from printouts took typically around 3 days including data entry and was liable to errors and inconsistencies.

With these things in mind we developed a second interactive KNIME workflow which takes the output of the library design workflow, and a number of other user inputs (scanned barcodes for reagent and reaction tubes, reagent tube tare weights and weights with compound added, reagent batch index used, the nature of any blanks incorporated, and the desired LCMS analysis method) and subsequently prepares all the input for the ELN which can be directly imported into an appropriate template writeup, along with a table of dilution volumes for the reagent plate.

The workflow then polls the LCMS data repository at 5-minute intervals until all samples have been analysed, and performs the analysis of the LCMS data automatically. The data can then simple be pasted into the ELN, and any manual edits made in the ELN and saved. The workflow retrieves the final, possibly edited, form of the QC data from the ELN, and generates a product table for import into the ELN with the correct fields for direct registration. This process can be completed in considerably less than 30 minutes for most libraries. The process is now sufficiently reliable than in our last library campaign (data not published), we found no CRMs where the automated protocol could not find product, where we subsequently could, and a random subset shown no mis-assigned products.

Finally, the workflow produces a platemap, along with graphical summaries of compound purity.

Without the CRM process, we would not have developed these protocols, as libraries were made in small batches of e.g. 6, 8 or 12 compounds, where the bottlenecks are less obvious. Even assuming that pure compounds are made in batches of 8 compounds using this protocol, the time used is increased by a factor of 12 during the library synthesis. If the protocol is not used, then the time is obviously increased considerably further.

Supplementary Note 3 - Trying to assign SM or PR automatically

The processing of the crystallographic datasets by PanDDA of CRMs soaked into protein crystals results in an “event map” of the electron density that is not present in an average map calculated by PanDDA for protein crystals only soaked with solvent. A manual inspection of the map assigns it as native or containing a ligand – either SM or PR. The ligand is fitted manually to this density with COOT[6] and refinement continues in PanDDA. PanDDA finishes with a calculation of how well the ligand fits the electron density using the CCP4 program EDSTATS[7]. We investigated whether the real space correlation coefficient (RSCC) could be used automatically to differentiate between SM and PR.

The real-space correlation coefficient (RSCC) is defined as:

$$RSCC = cov(\rho obs,\rho calc) / sqrt(var(\rho obs)\ var(\rho calc))$$

where cov(.,.) and var(.) are the sample covariance and variance (i.e. calculated with respect to the sample means of ρobs and ρcalc).

The first set of tests used the refined structures for Hsp90 shown in Figure 1 of the manuscript.

For the map of SM1 bound to Hsp90 (1.27Å resolution), the refined structure for:

(a) SM1 bound gave an RSCC of 0.95
(b) The product from CRM4 fitted gave an RSCC of 0.87

In this case, RSCC correctly identifies that SM1 is bound

For the map of SM2 bound to Hsp90 (1.16Å resolution), the refined structure for:

(a) SM1 bound gave an RSCC of 0.97
(b) SM2 bound gave an RSCC of 0.98
(c) The product from CRM4 fitted gave an RSCC of 0.95

In this case, RSCC is hardly distinguishing between something smaller (SM1) and something larger (product of CRM4) fitted to the density for SM2, even for such a high resolution structure

For the map of the product from CRM56 (compound **16**) which is derived from SM2, bound to PDHK2 (2.74 Å resolution), the refined structure for:

(a) SM2 bound gave an RSCC of 0.94

(b) Compound **16** bound gave an RSCC of 0.93

In this case, RSCC correctly identifies PR but there is very little difference to the value obtained for fitting SM

For the map of the product from CRM70 which is derived from SM2, bound to PDHK2 (2.04 Å resolution), the refined structure for:

(a) SM2 bound gave an RSCC of 0.99

(b) Product bound gave an RSCC of 0.97

In this case, RSCC incorrectly identifies SM but there is very little difference to the value obtained for fitting PR

It is not possible to use the fit to density metric, RSCC, to automatically identify whether starting material or product is bound when a crystal is soaked with a crude reaction mixture. For this reason, the experiments reported in the main text used manual inspection to assign the density, followed by manual fitting of the ligand before submitting to final refinement in PanDDA.

Supplementary Methods: Compound synthesis and characterisation

LCMS method to determine product yields in CRMs

Nominal 20 mM CRM stock solution in DMSO-d_6 was diluted (1 µL in 35 µL DMSO) and subjected to High performance liquid chromatography-high resolution mass spectroscopy (LC-MS; HRMS) analysis on an Agilent HP1290 system (Agilent HP1290 Infinity II series with Agilent TOF6230 single quadrupole mass spectrometer with an ESI source; Injection Volume: 1 µL). UV detection was at 230 nm, 254 nm and 270 nm.

Column:

Kinetex, 2.5 µm, C18, 50 ×2.1 mm (Phenomenex); Temperature: 55°C.

Mobile Phase:

A - H_2O + 10 mmol / ammonium formate + 0.08% (v/v) formic acid at pH ca 3.5.
B - 95% Acetonitrile + 5% A + 0.08% (v/v) formic acid.

Gradient:

Time (min)	Solvent A (%)	Solvent B (%)	Flow (mL/min)
0	95	5	1.3
0.12	95	5	1.3
1.30	5	95	1.3
1.35	5	95	1.6
1.85	5	95	1.6
1.90	5	95	1.3
1.95	95	5	1.3

High resolution mass spectroscopy (HRMS) analysis was performed on an Agilent HP1290 system (Agilent HP1290 Infinity II series with Agilent TOF6230 single quadrupole mass spectrometer with an ESI source; Injection Volume: 1 µL) under positive ionisation. All other conditions were as described above.

The LC-MS results were analysed automatically using a custom KNIME[8-11] (see also KNIME: Open for Innovation. https://www.knime.com/ (Accessed 2019-Aug-14)) workflow as follows:

1. UV (230, 254 and 270 nM) and MSD (+ve and -ve modes) relative peak areas were recalculated removing any peaks with a rt < 0.22 mins, in order to ignore the injection peak in the purity calculation
2. Any MSD peaks corresponding to product M^+, $[M+H]^+$, M^- or $[M-H]^-$ were identified, and the corresponding UV peaks identified
    a. In the event of multiple peaks matching, those with the smallest deviation from the target mass were preferred, followed by +ve ionisation mode over -ve ionisation mode, and greatest ionisation intensity
3. The rt of any MSD peaks corresponding to unreacted core starting material was identified as for step 2 above.
4. The purities for all traces where the corresponding peak was identified (2 × MSD, 3 × UV) were used to assign a purity category (>95%, 90-95%, 85-90%, <85%, UNKNOWN, No Product Observed) as follows.

a. Where the peak rt is overlapped with the unreacted SM retention time, the sample is assigned UNKNOWN. Any such samples were manually inspected to determine whether a purity could be assigned manually.
b. Where all purities fall within a single category, the sample is assigned to that category
c. Where the range of the mean purity ± 2 × std dev falls within a single category, the sample is assigned to that category.
d. For any remaining samples, a leave-one-out approach is applied, leaving out one purity (UV or MSD) in turn. The best purity obtained using this method is reported if it fulfils either of the criteria in b or c above
e. Remaining samples were analysed manually

5. The mean purity is reported in Table S1. Where leave-one-out was used to assign, the resulting mean was reported.

Experimental details for generation of all the CRM libraries

Compounds in the CRM libraries were synthesized following the general procedures A and B described previously from the corresponding amines as indicated below[4].

General Procedure A (SM1)

A 1.0 mL polypropylene Matrix storage tube was charged with **4** (0.05 M in DCM; 200 µL, 10 µmol). Triethylamine (7.0 µL, 5.0 mg, 50 µmol, 5.0 equiv) was added followed by the appropriate amine (1.0 M in DMF; 12.0 µL, 12.0 µmol, 1.2 equiv). The tube was capped, agitated briefly to ensure mixing, and allowed to stand at ambient temperature for 26 h. Ammonia (7.0 N in MeOH; 450 µL, 3.0 mmol, 300 equiv) was added and the tube recapped, agitated briefly to ensure mixing. After 48 h, the solvents were removed in vacuo (Genevac EZ-2; low bp mixture program; T_{max} 45 °C). The crude product was dissolved in DMSO-d6 (500 µL) to give a nominal 20 mM solution of CRM.

General Procedure B (SM2)

As for General Procedure A, substituting compound **9** in place of **4**.

General Procedure C – Lithium hydroxide hydrolysis of Acetate protecting groups and ester groups to free carboxylic acids (SM1)

As for general procedure A, except the ammonia deprotection step conditions were replaced with the following conditions in order to hydrolyse the methyl or ethyl ester to the free carboxylic acid:

Lithium Hydroxide (0.5 M in 1:1 MeOH-H_2O; 100 µL, 50.0 µmol, 5.0 equiv.) was added and the tube recapped, agitated briefly to ensure mixing. After 5 h, glacial acetic acid (20 µL) was added and the solvents were removed *in vacuo* (Genevac EZ2; med-low bp mixture program; T_{max} 45 °C). The crude product was dissolved in DMSO-d_6 (500 µL) to give a nominal 20 mM solution of CRM.

General Procedure D – Lithium hydroxide hydrolysis of Acetate protecting groups and ester groups to free carboxylic acids (SM2)
As for General Procedure C, substituting compound **9** in place of **4**.

CRMs from SM1

*2,4-Dihydroxy-*N*-methyl-*N*-phenylbenzamide (**CRM-1**).*
Prepared following General Procedure A, utilizing *N*-methylaniline: LCMS t_R = 1.04 min; *m/z* = 244.0 $[M+H]^+$; LC purity = 22.6%.

*4-(6-Fluoro-2-methyl-1,2,3,4-tetrahydroquinoline-1-carbonyl)benzene-1,3-diol (**CRM-2**).*
Prepared following General Procedure A, utilizing 6-fluoro-2-methyl-1,2,3,4-tetrahydroquinoline: LCMS t_R = 1.24 min; *m/z* = 301.8 $[M+H]^+$; LC purity = 8.0%.

*2,4-Dihydroxy-*N*-methyl-*N*-(2-methylphenyl)benzamide (**CRM-3**).*
Prepared following General Procedure A, utilizing *N*,2-dimethylaniline: LCMS t_R = 1.15 min; *m/z* = 258.0 $[M+H]^+$; LC purity = 8.0%.

*2,4-Dihydroxy-*N*-(4-hydroxyphenyl)-*N*-phenylbenzamide (**CRM-4**).*
Prepared following General Procedure A, utilizing 4-(phenylamino)phenol: LCMS t_R = 1.30 min; *m/z* = 322.0 $[M+H]^+$; LC purity = 42.4%.

*2-(*N*-Methyl-2,4-dihydroxybenzamido)benzoic acid (**CRM-5**).*
Prepared following General Procedure C, utilizing methyl 2-(methylamino)benzoate: LCMS t_R = 1.23 min; *m/z* = 274.0; LC purity = 50.0%.

N*-(5-Acetyl-4-methyl-1,3-thiazol-2-yl)-*N*-cyclopropyl-2,4-dihydroxybenzamide (**CRM-6**).*
Prepared following General Procedure A, utilizing 1-[2-(cyclopropylamino)-4-methyl-1,3-thiazol-5-yl]ethan-1-one: LCMS t_R = 1.00 min; *m/z* = 331.0 $[M-H]^-$; LC purity = 12.0%.

*2,4-Dihydroxy-*N*-(3-hydroxyphenyl)-*N*-(2-methylphenyl)benzamide (**CRM-7**).*
Prepared following General Procedure A, utilizing 3-[(2-methylphenyl)amino]phenol: LCMS t_R = 1.36 min; *m/z* = 336.0 $[M+H]^+$; LC purity = 48.1%.

N*-(4-Chlorophenyl)-2,4-dihydroxy-*N*-methylbenzamide (**CRM-8**).*
Prepared following General Procedure A, utilizing 4-chloro-*N*-methylaniline: LCMS t_R = 1.12 min; *m/z* = 278.0 $[M+H]^+$; LC purity = 28.0%.

*Methyl 2-(*N*-methyl-2,4-dihydroxybenzamido)benzoate (**CRM-9**).*
Prepared following General Procedure A, utilizing methyl 2-(methylamino)benzoate: LCMS t_R = 1.37 min; *m/z* = 302.0 $[M+H]^+$; LC purity = 1.1%.

*2,4-Dihydroxy-*N*-(3-methoxyphenyl)-*N*-methylbenzamide (**CRM-10**).*
Prepared following General Procedure A, utilizing 3-methoxy-*N*-methylaniline: LCMS t_R = 1.09 min; *m/z* = 273.8 $[M+H]^+$; LC purity = 14.0%.

*4-[6,7-Dimethoxy-1-(pyridin-3-yl)-1,2,3,4-tetrahydroisoquinoline-2-carbonyl]benzene-1,3-diol (**CRM-11**).*
Prepared following General Procedure A, utilizing 6,7-dimethoxy-1-(pyridin-3-yl)-1,2,3,4-tetrahydroisoquinoline: LCMS t_R = 0.86 min; *m/z* = 407.0 $[M+H]^+$; LC purity = 34.0%.

*4-(2,4-Dihydroxybenzoyl)-3,4-dihydro-2*H*-1,4-benzoxazine-2-carboxylic acid (**CRM-12**).*
Prepared following General Procedure C, utilizing ethyl 3,4-dihydro-2*H*-1,4-benzoxazine-2-carboxylate: LCMS t_R = 0.82 min; *m/z* = 314.0 $[M-H]^-$; LC purity = 8.7%.

*4-[(2*S*)-2-(Hydroxymethyl)-2,3-dihydro-1*H*-indole-1-carbonyl]benzene-1,3-diol (**CRM-13**).*
Prepared following General Procedure A, utilizing [(2*S*)-2,3-dihydro-1*H*-indol-2-yl]methanol: LCMS t_R = 1.17 min; *m/z* = 286.0 $[M+H]^+$; LC purity = 13.7%.

*4-{1'-Methyl-[2,4'-bipiperidine]-1-carbonyl}benzene-1,3-diol (**CRM-14**).*
Prepared following General Procedure A, utilizing 1'-methyl-2,4'-bipiperidine: LCMS t_R = 0.66 min; *m/z* = 319.0 $[M+H]^+$; LC purity = 28.0%.

*2,4-Dihydroxy-*N*-(4-methoxyphenyl)-*N*-methylbenzamide (**CRM-15**).*
Prepared following General Procedure A, utilizing 4-methoxy-*N*-methylaniline: LCMS t_R = 1.25 min; *m/z* = 274.0 $[M+H]^+$; LC purity = 1.0%.

N*-(2,4-Dichlorophenyl)-2,4-dihydroxy-*N*-methylbenzamide (**CRM-16**).*
Prepared following General Procedure A, utilizing 2,4-dichloro-*N*-methylaniline: LCMS Product not observed; LC purity = 0.0%.

N*-Ethyl-2,4-dihydroxy-*N*-(4-methylphenyl)benzamide (**CRM-17**).*
Prepared following General Procedure A, utilizing *N*-ethyl-4-methylaniline: LCMS t_R = 1.20 min; *m/z* = 272.0 $[M+H]^+$; LC purity = 5.5%.

*4-[7-(Trifluoromethyl)-4*H*,5*H*,6*H*,7*H*-pyrazolo[1,5-*a*]pyrimidine-4-carbonyl]benzene-1,3-diol (**CRM-18**).*
Prepared following General Procedure A, utilizing 7-(trifluoromethyl)-4*H*,5*H*,6*H*,7*H*-pyrazolo[1,5-a]pyrimidine: LCMS t_R = 0.64 min; *m/z* = 326.0 $[M-H]^-$; LC purity = 15.5%.

*1-(2,4-Dihydroxybenzoyl)piperidine-4-carboxylic acid (**CRM-19**).*
Prepared following General Procedure C, utilizing ethyl piperidine-4-carboxylate: LCMS t_R = 0.84 min; *m/z* = 279.8 $[M+MeOH]^+$; LC purity = 50.0%.

*2,4-Dihydroxy-*N*-(2-hydroxyethyl)-*N*-(1-methyl-1*H*-1,3-benzodiazol-2-yl)benzamide (**CRM-20**).*
Prepared following General Procedure A, utilizing 2-[(1-methyl-1*H*-1,3-benzodiazol-2-yl)amino]ethan-1-ol: LCMS t_R = 0.77 min; *m/z* = 328.0 $[M+H]^+$; LC purity = 48.1%.

*4-{2,8-Dimethyl-1*H*,2*H*,3*H*,4*H*,4a*H*,5*H*,9b*H*-pyrido[4,3-*b*]indole-5-carbonyl}benzene-1,3-diol (**CRM-21**).*

Prepared following General Procedure A, utilizing 2,8-dimethyl-1*H*,2*H*,3*H*,4*H*,4a*H*,5*H*,9b*H*-pyrido[4,3-*b*]indole: LCMS t_R = 0.71 min; *m/z* = 339.0 [M+H]$^+$; LC purity = 50.0%.

*2,4-Dihydroxy-*N*-methyl-*N*-(1-methylpiperidin-4-yl)benzamide (**CRM-22**).*
Prepared following General Procedure A, utilizing *N*,1-dimethylpiperidin-4-amine: LCMS t_R = 0.21 min; *m/z* = 265.0 [M+H]$^+$; LC purity = 25.0%.

N*-(3-Fluorophenyl)-2,4-dihydroxy-*N*-methylbenzamide (**CRM-23**).*
Prepared following General Procedure A, utilizing 3-fluoro-*N*-methylaniline: LCMS Product not observed; LC purity = 0.0%.

*4-(3,4-Dihydro-2*H*-1,4-benzoxazine-4-carbonyl)benzene-1,3-diol (**CRM-24**).*
Prepared following General Procedure A, utilizing 3,4-dihydro-2*H*-1,4-benzoxazine: LCMS t_R = 1.18 min; *m/z* = 273.0 [M+H]$^+$; LC purity = 1.0%.

N*-(7-Chloro-2*H*-1,3-benzodioxol-5-yl)-2,4-dihydroxy-*N*-methylbenzamide (**CRM-25**).*
Prepared following General Procedure A, utilizing 7-chloro-*N*-methyl-2*H*-1,3-benzodioxol-5-amine: LCMS t_R = 1.10 min; *m/z* = 322.0 [M+H]$^+$; LC purity = 62.1%.

*2,4-Dihydroxybenzamide (**CRM-26***).*
Prepared following General Procedure A, utilizing ammonia (7.0 M in MeOH): LCMS t_R = 0.28 min; *m/z* = 154.0 [M+H]$^+$; LC purity = 74.4%.

*2,4-Dihydroxy-*N*-methyl-*N*-[2-(morpholin-4-yl)-1-phenylethyl]benzamide (**CRM-27**).*
Prepared following General Procedure A, utilizing methyl[2-(morpholin-4-yl)-1-phenylethyl]amine: LCMS t_R = 0.79 min; *m/z* = 357.0 [M+H]$^+$; LC purity = 28.1%.

*2-(2,4-Dihydroxybenzoyl)-1,2,3,4-tetrahydroisoquinoline-7-carbonitrile (**CRM-28**).*
Prepared following General Procedure A, utilizing 1,2,3,4-tetrahydroisoquinoline-7-carbonitrile: LCMS t_R = 0.94 min; *m/z* = 295.0 [M+H]$^+$; LC purity = 38.0%.

N*-Cyclohexyl-2,4-dihydroxy-*N*-(2-hydroxyethyl)benzamide (**CRM-29**).*
Prepared following General Procedure A, utilizing 2-(cyclohexylamino)ethan-1-ol: LCMS t_R = 0.87 min; *m/z* = 280.0 [M+H]$^+$; LC purity = 3.9%.

N,N*-Dicyclohexyl-2,4-dihydroxybenzamide (**CRM-30**).*
Prepared following General Procedure A, utilizing *N*-cyclohexylcyclohexanamine: LCMS Product not observed; LC purity = 0.0%.

*4-(5-Methyl-2,3-dihydro-1*H*-indole-1-carbonyl)benzene-1,3-diol (**CRM-31**).*
Prepared following General Procedure A, utilizing 5-methyl-2,3-dihydro-1*H*-indole: LCMS Product not observed; LC purity = 0.0%.

*4-(6-Methyl-1,2,3,4-tetrahydroquinoline-1-carbonyl)benzene-1,3-diol (**CRM-32**).*
Prepared following General Procedure A, utilizing 6-methyl-1,2,3,4-tetrahydroquinoline: LCMS t_R = 1.14 min; *m/z* = 284.0 [M+H]$^+$; LC purity = 42.7%.

*1-(2,4-Dihydroxybenzoyl)-*N,N*-dimethyl-2,3-dihydro-1*H*-indole-5-sulfonamide (**CRM-33**).*

Prepared following General Procedure A, utilizing *N*,*N*-dimethyl-2,3-dihydro-1*H*-indole-5-sulfonamide: LCMS t_R = 0.98 min; *m/z* = 363.0 $[M+H]^+$; LC purity = 44.0%.

*4-{3-[4-(Trifluoromethyl)phenoxy]piperidine-1-carbonyl}benzene-1,3-diol (**CRM-34**).*
Prepared following General Procedure A, utilizing 3-[4-(trifluoromethyl)phenoxy]piperidine: LCMS t_R = 1.19 min; *m/z* = 382.0 $[M+H]^+$; LC purity = 82.0%.

*5-(2,4-Dihydroxybenzoyl)-4-(ethoxycarbonyl)-3*H*,3a*H*,4*H*,5*H*,9b*H*-cyclopenta[*c*]quinoline-8-carboxylic acid (**CRM-35**).*
Prepared following General Procedure A, utilizing 4-(ethoxycarbonyl)-3*H*,3a*H*,4*H*,5*H*,9b*H*-cyclopenta[*c*]quinoline-8-carboxylic acid: LCMS Product not observed; LC purity = 0.0%.

*4-(1,2,3,4-Tetrahydroquinoline-1-carbonyl)benzene-1,3-diol (**CRM-36**).*
Prepared following General Procedure A, utilizing 1,2,3,4-tetrahydroquinoline: LCMS Product not observed; LC purity = 0.0%.

*4-(2,3-Dihydro-1*H*-indole-1-carbonyl)benzene-1,3-diol (**CRM-37**).*
Prepared following General Procedure A, utilizing 2,3-dihydro-1*H*-indole: LCMS t_R = 1.01 min; *m/z* = 256.0 $[M+H]^+$; LC purity = 4.0%.

N*-(4-Bromophenyl)-2,4-dihydroxy-*N*-methylbenzamide (**CRM-38**).*
Prepared following General Procedure A, utilizing 4-bromo-*N*-methylaniline: LCMS t_R = 1.10 min; *m/z* = 321.8 $[M+H]^+$; LC purity = 12.0%.

*2,4-Dihydroxy-*N*-(2-hydroxyethyl)-*N*-[1-(2-oxo-2-phenylethyl)-1*H*-1,3-benzodiazol-2-yl]benzamide (**CRM-39**).*
Prepared following General Procedure A, utilizing 2-{2-[(2-hydroxyethyl)amino]-1*H*-1,3-benzodiazol-1-yl}-1-phenylethan-1-one: LCMS t_R = 0.92 min; *m/z* = 432.0 $[M+H]^+$; LC purity = 37.0%.

*2,4-Dihydroxy-*N*-(2-methoxyethyl)-*N*-[5-(trifluoromethyl)pyridin-2-yl]benzamide (**CRM-40**).*
Prepared following General Procedure A, utilizing *N*-(2-methoxyethyl)-5-(trifluoromethyl)pyridin-2-amine: LCMS t_R = 1.09 min; *m/z* = 357.0 $[M+H]^+$; LC purity = 39.0%.

*2,4-Dihydroxy-*N*-(2-hydroxyphenyl)-*N*-methylbenzamide (**CRM-41**).*
Prepared following General Procedure A, utilizing 2-(methylamino)phenol: LCMS t_R = 0.88 min; *m/z* = 260.0 $[M+H]^+$; LC purity = 10.0%.

*4-[1-(4-Ethoxyphenyl)-1*H*,2*H*,3*H*,4*H*-pyrrolo[1,2-*a*]pyrazine-2-carbonyl]benzene-1,3-diol (**CRM-42**).*
Prepared following General Procedure A, utilizing 1-(4-ethoxyphenyl)-1*H*,2*H*,3*H*,4*H*-pyrrolo[1,2-*a*]pyrazine: LCMS t_R = 1.18 min; *m/z* = 379.0 $[M+H]^+$; LC purity = 10.0%.

N*-Benzyl-2,4-dihydroxy-*N*-phenylbenzamide (**CRM-43**).*
Prepared following General Procedure A, utilizing *N*-benzylaniline: LCMS t_R = 1.27 min; *m/z* = 320.0 $[M+H]^+$; LC purity = 19.0%.

*4-(6-Methyl-1,2,3,4-tetrahydroquinoline-1-carbonyl)benzene-1,3-diol (**CRM-44**).*
Prepared following General Procedure A, utilizing 6-methyl-1,2,3,4-tetrahydroquinoline: LCMS t_R = 1.14 min; *m/z* = 284.0 $[M+H]^+$; LC purity = 1.7%.

*Benzyl 4-(2,4-dihydroxybenzoyl)piperazine-1-carboxylate (**CRM-45**).*
Prepared following General Procedure A, utilizing benzyl piperazine-1-carboxylate: LCMS t_R = 1.02 min; *m/z* = 357.0 $[M+H]^+$; LC purity = 29.0%.

N-*Benzyl-2,4-dihydroxy-*N-*(pyridin-2-yl)benzamide (**CRM-46**).*
Prepared following General Procedure A, utilizing *N*-benzylpyridin-2-amine: LCMS t_R = 1.09 min; *m/z* = 321.0 $[M+H]^+$; LC purity = 35.0%.

N-*[4-(1,3-Benzothiazol-2-yl)phenyl]-2,4-dihydroxy-*N-*methylbenzamide (**CRM-47**).*
Prepared following General Procedure A, utilizing 4-(1,3-benzothiazol-2-yl)-*N*-methylaniline: LCMS t_R = 1.24 min; *m/z* = 377.0 $[M+H]^+$; LC purity = 48.0%.

*4-(6,7-Dimethoxy-1-phenyl-1,2,3,4-tetrahydroisoquinoline-2-carbonyl)benzene-1,3-diol (**CRM-48**).*
Prepared following General Procedure A, utilizing 6,7-dimethoxy-1-phenyl-1,2,3,4-tetrahydroisoquinoline: LCMS t_R = 1.11 min; *m/z* = 406.0 $[M+H]^+$; LC purity = 82.0%.

*2,4-Dihydroxy-*N-*[(1*S*,2*S*)-1-hydroxy-1-(4-methylphenyl)propan-2-yl]-*N-*(propan-2-yl)benzamide (**CRM-49**).*
Prepared following General Procedure A, utilizing (1*S*,2*S*)-1-(4-methylphenyl)-2-[(propan-2-yl)amino]propan-1-ol: LCMS t_R = 0.94 min; *m/z* = 344.0 $[M+H]^+$; LC purity = 15.0%.

N-*Benzyl-2,4-dihydroxy-*N-*(4-methylpyridin-2-yl)benzamide (**CRM-50**).*
Prepared following General Procedure A, utilizing *N*-benzyl-4-methylpyridin-2-amine: LCMS t_R = 1.21 min; *m/z* = 335.0 $[M+H]^+$; LC purity = 30.0%.

*Ethyl 4-(2,4-dihydroxybenzoyl)-3,4-dihydro-2*H*-1,4-benzoxazine-2-carboxylate (**CRM-51**).*
Prepared following General Procedure A, utilizing ethyl 3,4-dihydro-2*H*-1,4-benzoxazine-2-carboxylate: LCMS Product not observed; LC purity = 0.0%.

*Ethyl 1-(2,4-dihydroxybenzoyl)piperidine-4-carboxylate (**CRM-52**).*
Prepared following General Procedure A, utilizing ethyl piperidine-4-carboxylate: LCMS t_R = 0.97 min; *m/z* = 293.8 $[M+H]^+$; LC purity = 7.0%.

N-*(1,1-Dioxo-1λ^6-thiolan-3-yl)-*N-*ethyl-2,4-dihydroxybenzamide (**CRM-53**).*
Prepared following General Procedure A, utilizing 3-(ethylamino)-1λ^6-thiolane-1,1-dione: LCMS t_R = 0.71 min; *m/z* = 298.0 $[M-H]^-$; LC purity = 7.0%.

*4-[3-(Hydroxymethyl)-1,2,3,4-tetrahydroisoquinoline-2-carbonyl]benzene-1,3-diol (**CRM-54**).*
Prepared following General Procedure A, utilizing (1,2,3,4-tetrahydroisoquinolin-3-yl)methanol: LCMS t_R = 0.88 min; *m/z* = 300.0 $[M+H]^+$; LC purity = 100.0%.

*2,4-Dihydroxy-*N,N-*bis(3-hydroxyphenyl)benzamide (**CRM-55**).*

Prepared following General Procedure A, utilizing 3-[(3-hydroxyphenyl)amino]phenol: LCMS t_R = 1.13 min; *m/z* = 336.0 $[M-H]^-$; LC purity = 36.1%.

CRMs from SM2

*2,4-Dihydroxy-*N*-methyl-i-{4-[methyl(phenyl)carbamoyl]phenyl}benzamide (**CRM-56**).*
Prepared following General Procedure B, utilizing *N*-methylaniline: LCMS t_R = 1.08 min; *m/z* = 377.0 $[M+H]^+$; LC purity = 84.5%.

N*-[4-(6-Fluoro-2-methyl-1,2,3,4-tetrahydroquinoline-1-carbonyl)phenyl]-2,4-dihydroxy-N-methylbenzamide (**CRM-57**).*
Prepared following General Procedure B, utilizing 6-fluoro-2-methyl-1,2,3,4-tetrahydroquinoline: LCMS t_R = 1.23 min; *m/z* = 435.0 $[M+H]^+$; LC purity = 39.0%.

*2,4-Dihydroxy-*N*-methyl-*N*-{4-[methyl(2-methylphenyl)carbamoyl]phenyl}benzamide (**CRM-58**).*
Prepared following General Procedure B, utilizing *N*,2-dimethylaniline: LCMS t_R = 1.12 min; *m/z* = 391.0 $[M+H]^+$; LC purity = 100.0%.

*2-[*N*-Methyl4-(N-methyl2,4-dihydroxybenzamido)benzamido]benzoic acid (**CRM-59**).*
Prepared following General Procedure D, utilizing methyl 2-(methylamino)benzoate: LCMS Product not observed; LC purity = 0.0%.

N*-{4-[(4-Chlorophenyl)(methyl)carbamoyl]phenyl}-2,4-dihydroxy-*N*-methylbenzamide (**CRM-60**).*
Prepared following General Procedure B, utilizing 4-chloro-*N*-methylaniline: LCMS t_R = 1.16 min; *m/z* = 411.0 $[M+H]^+$; LC purity = 100.0%.

*Methyl 2-[N-methyl4-(N-methyl2,4-dihydroxybenzamido)benzamido]benzoate (**CRM-61**).*
Prepared following General Procedure B, utilizing methyl 2-(methylamino)benzoate: LCMS t_R = 1.06 min; *m/z* = 435.0 $[M+H]^+$; LC purity = 27.0%.

*2,4-Dihydroxy-*N*-{4-[(3-methoxyphenyl)(methyl)carbamoyl]phenyl}-*N*-methylbenzamide (**CRM-62**).*
Prepared following General Procedure B, utilizing 3-methoxy-*N*-methylaniline: LCMS t_R = 1.11 min; *m/z* = 407.0 $[M+H]^+$; LC purity = 76.9%.

*4-[4-(*N*-Methyl-2,4-dihydroxybenzamido)benzoyl]-3,4-dihydro-2*H*-1,4-benzoxazine-2-carboxylic acid (**CRM-63**).*
Prepared following General Procedure D, utilizing ethyl 3,4-dihydro-2*H*-1,4-benzoxazine-2-carboxylate: LCMS t_R = 0.97 min; *m/z* = 448.8 $[M+H]^+$; LC purity = 8.7%.

*2,4-Dihydroxy-*N*-{4-[(4-methoxyphenyl)(methyl)carbamoyl]phenyl}-*N*-methylbenzamide (**CRM-64**).*
Prepared following General Procedure B, utilizing 4-methoxy-*N*-methylaniline: LCMS t_R = 1.54 min; *m/z* = 405.8 $[M-H]^-$; LC purity = 9.0%.

N-*{4-[(2,4-Dichlorophenyl)(methyl)carbamoyl]phenyl}-2,4-dihydroxy*-N-*methylbenzamide (**CRM-65**).*
Prepared following General Procedure B, utilizing 2,4-dichloro-*N*-methylaniline: LCMS Product not observed; LC purity = 0.0%.

N-*{4-[Ethyl(4-methylphenyl)carbamoyl]phenyl}-2,4-dihydroxy*-N-*methylbenzamide (**CRM-66**).*
Prepared following General Procedure B, utilizing *N*-ethyl-4-methylaniline: LCMS t_R = 1.21 min; *m/z* = 405.0 $[M+H]^+$; LC purity = 100.0%.

*1-[4-(*N-*Methyl2,4-dihydroxybenzamido)benzoyl]piperidine-4-carboxylic acid (**CRM-67**).*
Prepared following General Procedure D, utilizing ethyl piperidine-4-carboxylate: LCMS t_R = 0.82 min; *m/z* = 399.0 $[M+H]^+$; LC purity = 71.6%.

2,4-Dihydroxy-N-*methyl*-N-*{4-[methyl(1-methylpiperidin-4-yl)carbamoyl]phenyl}benzamide (**CRM-68**).*
Prepared following General Procedure B, utilizing *N*,1-dimethylpiperidin-4-amine: LCMS t_R = 0.73 min; *m/z* = 398.0 $[M+H]^+$; LC purity = 8.8%.

N-*{4-[(3-Fluorophenyl)(methyl)carbamoyl]phenyl}-2,4-dihydroxy*-N-*methylbenzamide (**CRM-69**).*
Prepared following General Procedure B, utilizing 3-fluoro-*N*-methylaniline: LCMS t_R = 1.11 min; *m/z* = 395.0 $[M+H]^+$; LC purity = 90.0%.

N-*[4-(3,4-dihydro-2*H-*1,4-benzoxazine-4-carbonyl)phenyl]-2,4-dihydroxy*-N-*methylbenzamide (**CRM-70**).*
Prepared following General Procedure B, utilizing 3,4-dihydro-2*H*-1,4-benzoxazine: LCMS t_R = 1.13 min; *m/z* = 405.0 $[M+H]^+$; LC purity = 88.0%.

N-*(4-Carbamoylphenyl)-2,4-dihydroxy*-N-*methylbenzamide (**CRM-71**).*
Prepared following General Procedure B, utilizing ammonia (7 N in MeOH): LCMS t_R = 0.87 min; *m/z* = 288.0 $[M+H]^+$; LC purity = 78.0%.

N-*{4-[Cyclohexyl(2-hydroxyethyl)carbamoyl]phenyl}-2,4-dihydroxy*-N-*methylbenzamide (**CRM-72**).*
Prepared following General Procedure B, utilizing 2-(cyclohexylamino)ethan-1-ol: LCMS t_R = 1.03 min; *m/z* = 413.0 $[M+H]^+$; LC purity = 8.3%.

N-*[4-(Dicyclohexylcarbamoyl)phenyl]-2,4-dihydroxy*-N-*methylbenzamide (**CRM-73**).*
Prepared following General Procedure B, utilizing *N*-cyclohexylcyclohexanamine: LCMS t_R = 1.39 min; *m/z* = 451.0 $[M+H]^+$; LC purity = 1.0%.

2,4-Dihydroxy-N-*methyl*-N-*[4-(5-methyl-2,3-dihydro-1*H-*indole-1-carbonyl)phenyl]benzamide (**CRM-74**).*
Prepared following General Procedure B, utilizing 5-methyl-2,3-dihydro-1*H*-indole: LCMS Product not observed; LC purity = 0.0%.

2,4-Dihydroxy-N-*methyl*-N-*[4-(1,2,3,4-tetrahydroquinoline-1-carbonyl)phenyl]benzamide (**CRM-75**).*

Prepared following General Procedure B, utilizing 1,2,3,4-tetrahydroquinoline: LCMS t_R = 1.16 min; *m/z* = 403.0 $[M+H]^+$; LC purity = 3.3%.

N-*[4-(2,3-Dihydro-1*H-*indole-1-carbonyl)phenyl]-2,4-dihydroxy*-N-*methylbenzamide* *(**CRM-76**).*
Prepared following General Procedure B, utilizing 2,3-dihydro-1*H*-indole: LCMS t_R = 1.15 min; *m/z* = 389.0 $[M+H]^+$; LC purity = 43.0%.

N-*{4-[(4-Bromophenyl)(methyl)carbamoyl]phenyl}-2,4-dihydroxy*-N-*methylbenzamide* *(**CRM-77**).*
Prepared following General Procedure B, utilizing 4-bromo-*N*-methylaniline: LCMS t_R = 1.20 min; *m/z* = 455.0 $[M+H]^+$; LC purity = 53.0%.

N-*{4-[Benzyl(phenyl)carbamoyl]phenyl}-2,4-dihydroxy*-N-*methylbenzamide (**CRM-78**).*
Prepared following General Procedure B, utilizing *N*-benzylaniline: LCMS t_R = 1.28 min; *m/z* = 453.0 $[M+H]^+$; LC purity = 88.0%.

2,4-Dihydroxy-N-*methyl*-N-*[4-(6-methyl-1,2,3,4-tetrahydroquinoline-1-carbonyl)phenyl]benzamide (**CRM-79**).*
Prepared following General Procedure B, utilizing 6-methyl-1,2,3,4-tetrahydroquinoline: LCMS t_R = 1.22 min; *m/z* = 417.0 $[M+H]^+$; LC purity = 30.0%.

*Benzyl 4-[4-(*N-*methyl2,4-dihydroxybenzamido)benzoyl]piperazine-1-carboxylate (**CRM-80**).*
Prepared following General Procedure B, utilizing benzyl piperazine-1-carboxylate: LCMS t_R = 1.16 min; *m/z* = 490.0 $[M+H]^+$; LC purity = 37.0%.

N-*{4-[Benzyl(4-methylpyridin-2-yl)carbamoyl]phenyl}-2,4-dihydroxy*-N-*methylbenzamide (**CRM-81**).*
Prepared following General Procedure B, utilizing *N*-benzyl-4-methylpyridin-2-amine: LCMS t_R = 1.22 min; *m/z* = 468.0 $[M+H]^+$; LC purity = 10.0%.

*Ethyl 4-[4-(*N-*methyl2,4-dihydroxybenzamido)benzoyl]-3,4-dihydro-2*H-*1,4-benzoxazine-2-carboxylate (**CRM-82**).*
Prepared following General Procedure B, utilizing ethyl 3,4-dihydro-2*H*-1,4-benzoxazine-2-carboxylate: LCMS t_R = 0.99 min; *m/z* = 475.8 $[M-H]^-$; LC purity = 27.0%.

*Ethyl 1-[4-(*N-*methyl2,4-dihydroxybenzamido)benzoyl]piperidine-4-carboxylate (**CRM-83**).*
Prepared following General Procedure B, utilizing ethyl piperidine-4-carboxylate: LCMS t_R = 1.08 min; *m/z* = 427.0 $[M+H]^+$; LC purity = 8.0%.

Synthesis of individual compounds listed in the paper

2,4-Dihydroxybenzamide (***1***). A pure sample was purchased from Apollo Scientific (https://www.apolloscientific.co.uk/; Cat. No. OR-0447)

2,4-Bis(acetoxy)benzoic acid (***4***) was synthesized according to the route shown in Figure 2 of the main manuscript following the previously reported procedure[4].

*4-[*N*-Methyl-2,4-bis(acetoxy)benzamido]benzoic acid* (***9***) was synthesized according to the route shown in Figure 2 of the main manuscript following the previously reported procedure[4].

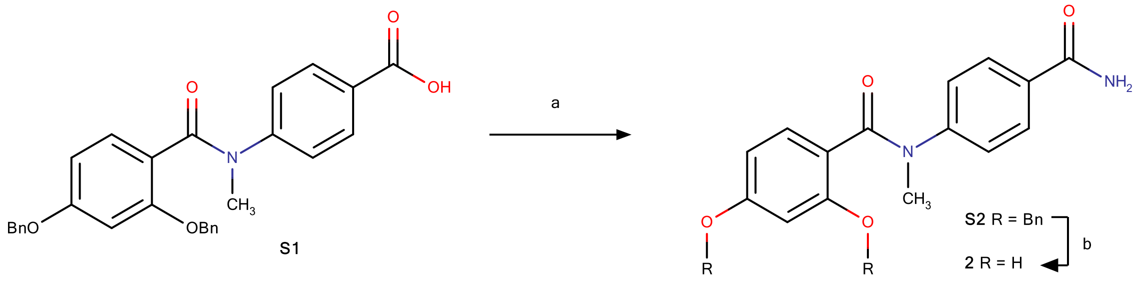

Supplementary Figure 7. Scheme for synthesis of **2**. *Reagents and conditions:* a. i. EDCI, HOBt-NH_3, Et_3N, THF, **S1**, 50 °C; b. H_2, Pd/C, MeOH-AcOH.

*4-[*N*-Methyl-2,4-bis(benzyloxy)benzamido]benzamide (**S2**)*
A suspension of methyl-2,4-bis(benzyloxy)benzamido]benzoic acid[4] **S1** (150 mg, 0.32 mmol), 1-hydroxybenzotriazole ammonia salt (97 mg, 0.64 mmol), 1-ethyl-3-(3-dimethylaminopropyl)carbodiimide (123 mg, 0.64 mmol) and triethylamine (134 µL, 97 mg, 0.96 mmol) in dry THF (5 mL) was heated to 50 °C whereupon a clear solution formed. The reaction was maintained at this temperature for 3.5 h, cooled to room temperature and diluted with ethyl acetate (50 mL) and washed successively with $NaHCO_3$ (sat. aq; 50 mL) and brine (50 mL). The organic layer was dried ($MgSO_4$) and the solvents removed *in vacuo*. The crude product was filtered through silica eluting with 10% MeOH-DCM to give the *amide* **S2** (83 mg, 56%) as a white solid; LCMS t_R = 1.26 min; m/z = 467.0 $[M+H]^+$; ^{1}H NMR (399 MHz, DMSO-d_6) δ 7.93 (s, 1H), 7.71 (d, J = 8.2 Hz, 2H), 7.48 – 7.29 (m, 10H), 7.14 (t, J = 7.8 Hz, 3H), 6.71 – 6.44 (m, 2H), 5.02 (s, 2H), 5.00 (s, 2H), 3.30 (s, 3H).

N-*(4-Carbamoylphenyl)-2,4-dihydroxy-*N*-methylbenzamide (**2**)*
A suspension of 4-[*N*-Methyl-2,4-bis(benzyloxy)benzamido]benzamide **S2** (83 mg, 0.18 mmol) in methanol (3 mL) and glacial acetic acid (3 mL) was added to a flask containing palladium (10% on carbon; 20 mg) under a nitrogen atmosphere. The atmosphere was replaced with a hydrogen atmosphere and shaken for 21 h. The reaction was filtered through celite and the solvent removed *in vacuo*. The crude product was purified by flash column chromatography (SiO_2) using a DCM to 5% MeOH-DCM gradient to give the product **2** as a white solid (44.1 mg, 86%); LCMS t_R = 0.77 min; m/z = 287.0 $[M+H]^+$; ^{1}H

NMR (399 MHz, DMSO-d_6) δ 9.87 (s, 1H), 9.60 (s, 1H), 7.92 (s, 1H), 7.82 – 7.66 (m, 2H), 7.35 (s, 1H), 7.26 – 7.13 (m, 2H), 6.82 (d, J = 8.4 Hz, 1H), 6.11 (d, J = 2.2 Hz, 1H), 6.06 (dd, J = 8.4, 2.2 Hz, 1H), 3.32 (s, 3H).

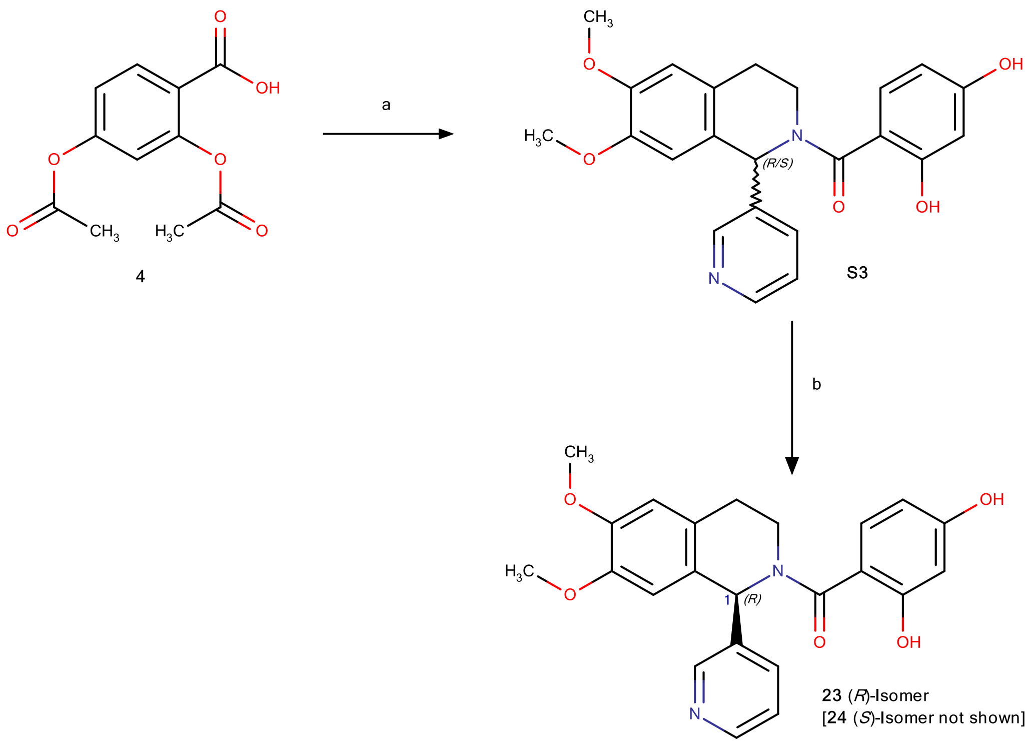

Supplementary Figure 8. Scheme for synthesis of **23** and **24** *Reagents and conditions:* a. i. $(COCl)_2$, DMF (cat.), DCM, **4**; ii. 6,7-dimethoxy-1-(pyridine-3-yl)-1,2,3,4-tetrahydroisoquinoline, Et_3N, DCM; iii. NH_3, MeOH; b. ChiralPak AD, 95% Hexane-IPA, 0.2% TFA.

rac-*4-{[6,7-Dimethoxy-1-(pyridin-3-yl)-1,2,3,4-tetrahydroisoquinolin-2-yl]carbonyl}benzene-1,3-diol (**S3**) – Supplementary Figure 8.*

A solution of Oxalyl chloride solution (2.0 M in DCM; 1.01 mL, 2 M, 2.03 mmol, 1.35 eq) was added drop-wise, at ambient temperature, under nitrogen atmosphere, to a solution of 2,4-bis(acetoxy)benzoic acid **4** (358 mg, 1.5 mmol, 1 eq) in DCM (13 mL) . Several drops of anhydrous DMF was added to the reaction mixture, immediately causing gas evolution and a homogeneous mixture. This solution was left to stir under nitrogen atmosphere for 2 h affording a yellow solution. The reaction mixture was evaporated *in vacuo* and then re-dissolved in DCM (20 mL) and evaporated again in vacuo. The resulting oil was dissolved in DCM (5 mL) under N_2, and solution cooled with an ice-water bath. A solution of 6,7-dimethoxy-1-(pyridin-3-yl)-1,2,3,4-tetrahydroisoquinoline (541.62 mg, 1.5 mmol, 1 eq) and triethylamine (0.94 mL, 6.76 mmol, 4.5 eq) in DCM (6 mL) was added to the acid chloride solution, stirred at 0°C for 30 mins then over weekend at RT. Ammonia (7 N in MeOH; 6.44 mL, 45.09 mmol, 30 eq) was added and mixture was stirred for 21 h at RT. The solvents were evaporated *in vacuo* and the crude product was dissolved in ethyl acetate (40 mL) and organic phase then washed sequentially with water (2 × 30 mL) then with brine (30 mL), filtered and evaporated *in vacuo* to a yellow gum which was purified by automated

flash chromatography on silica gel (CombiflashRf, 24g RediSep column) eluting with a solvent gradient of 0 to 8% MeOH in DCM. This afforded the *amide* (188 mg) which was re-purified by reverse phase chromatography (12 g, Grace C18), eluting with a solvent gradient of 10-100% MeCN in water to give the racemic *amide* (85 mg, 0.21 mmol, 13.9%) as a colorless powder; LCMS t_R = 0.89; *m/z* = 407 $[M+H]^+$; ^{1}H NMR (400 MHz, DMSO-d_6) δ 2.68 (dt, *J* = 4.1, 16.1 Hz, 1H), 2.88 (ddd, *J* = 6.0, 10.4, 16.4 Hz, 1H), 3.19 – 3.31 (m, 1H), 3.65 - 3.78 (m, 1H), 3.70 (s, 3H), 3.79 (s, 3H), 6.28 (dd, *J* = 2.2, 8.3 Hz, 1H), 6.34 (d, *J* = 2.2 Hz, 1H), 6.61 (s, 1H), 6.76 (s, 1H), 6.82 (s, 1H), 6.91 (d, *J* = 8.3 Hz, 1H), 7.31 (ddd, *J* = 0.9, 4.7, 7.9 Hz, 1H), 7.57 (dt, *J* = 2.1, 8.0 Hz, 1H), 8.40 – 8.53 (m, 2H), 9.44 (s, 2H).

*4-[(1*R*)-6,7-Dimethoxy-1-(pyridin-3-yl)-1,2,3,4-tetrahydroisoquinoline-2-carbonyl]benzene-1,3-diol (**23**)*
and
*4-[(1*S*)-6,7-Dimethoxy-1-(pyridin-3-yl)-1,2,3,4-tetrahydroisoquinoline-2-carbonyl]benzene-1,3-diol (**24**)*
An aliquot of *rac*-4-{[6,7-Dimethoxy-1-(pyridin-3-yl)-1,2,3,4-tetrahydroisoquinolin-2-yl]carbonyl}benzene-1,3-diol **S3** (XX mg, xx mmol) was separated by chiral chromatography (ChiralPak AD; 250 × 20 mm column; 10 micron particle size; 15 mL / min; 95% hexane-IPA containing 0.2% TFA; runtime 37 min) to give the *(R)-isomer* **23** (XX mg, xx%) as a white powder; LCMS t_R = XX; *m/z* = XX $[M+H]^+$; ^{1}H NMR (399 MHz, DMSO-d_6) δ 9.73 (s, 1H), 9.60 (s, 1H), 8.53 (s, 1H), 8.49 - 8.43 (m, 1H), 7.58 (s, 1H), 7.34 (t, J = 6.30 Hz, 1H), 6.91 (d, J = 8.20 Hz, 1H), 6.82 (s, 2H), 6.34 - 6.22 (m, 2H), 3.76 (s, 3H), 3.67 (s, 3H), 3.22 (s, 1H), 2.85 (ddd, J = 5.96, 10.54, 16.40 Hz, 1H), 2.66 (d, J = 16.35 Hz, 1H) and the *(S)-isomer* **24** (XX mg, xx%) as a white powder; LCMS t_R = XX; *m/z* = XX $[M+H]^+$; ^{1}H NMR (399 MHz, DMSO-d_6) δ 9.76 (s, 1H), 9.64 (s, 1H), 8.53 (s, 1H), 8.49 - 8.43 (m, 1H), 7.58 (s, 1H), 7.38 - 7.30 (m, 1H), 6.91 (d, J = 8.20 Hz, 1H), 6.82 (s, 2H), 6.35 - 6.22 (m, 2H), 3.76 (s, 3H), 3.67 (s, 3H), 3.47 (s, 1H), 3.20 (d, J = 15.19 Hz, 1H), 2.85 (ddd, J = 5.97, 10.52, 16.35 Hz, 1H), 2.71 - 2.61 (m, 1H), 1.24 (s, 1H).

Supplementary References

1. S. Roughley, L. Wright, P. Brough, A. Massey and R. E. Hubbard, in *Fragment-Based Drug Discovery and X-Ray Crystallography*, eds. T. G. Davies and M. Hyvonen, 2012, vol. 317, pp. 61-82.
2. J. D. Moore, A. Staniszewska, T. Shaw, J. D'Alessandro, B. Davis, A. Surgenor, L. Baker, N. Matassova, J. Murray, A. Macias, P. Brough, M. Wood and P. C. Mahon, *Oncotarget*, 2014, **5**, 12862-12876.
3. X. Barril, P. Brough, M. Drysdale, R. E. Hubbard, A. Massey, A. Surgenor and L. Wright, *Bioorg Med Chem Lett*, 2005, **15**, 5187-5191.
4. P. A. Brough, L. Baker, S. Bedford, K. Brown, S. Chavda, V. Chell, J. D'Alessandro, N. G. Davies, B. Davis, L. Le Strat, A. T. Macias, D. Maddox, P. C. Mahon, A. J. Massey, N. Matassova, S. McKenna, J. W. Meissner, J. D. Moore, J. B. Murray, C. J. Northfield, C. Parry, R. Parsons, S. D. Roughley, T. Shaw, H. Simmonite, S. Stokes, A. Surgenor, E. Stefaniak, A. Robertson, Y. Wang, P. Webb, N. Whitehead and M. Wood, *J Med Chem*, 2017, **60**, 2271-2286.
5. J. B. Murray, S. D. Roughley, N. Matassova and P. A. Brough, *J Med Chem*, 2014, **57**, 2845-2850.
6. P. Emsley and K. Cowtan, *Acta Crystallogr D Biol Crystallogr*, 2004, **60**, 2126-2132.
7. M. D. Winn, C. C. Ballard, K. D. Cowtan, E. J. Dodson, P. Emsley, P. R. Evans, R. M. Keegan, E. B. Krissinel, A. G. W. Leslie, A. McCoy, S. J. McNicholas, G. N. Murshudov, N. S. Pannu, E. A. Potterton, H. R. Powell, R. J. Read, A. Vagin and K. S. Wilson, *Acta Crystallographica Section D: Biological Crystallography*, 2011, **67**, 235-242.
8. M. R. Berthold, N. Cebron, F. Dill, T. R. Gabriel, T. K\, \#246, tter, T. Meinl, P. Ohl, K. Thiel and B. Wiswedel, *SIGKDD Explor. Newsl.*, 2009, **11**, 26-31.
9. M. R. Berthold, N. Cebron, F. Dill, T. R. Gabriel, T. Kötter, T. Meinl, P. Ohl, C. Sieb, K. Thiel and B. Wiswedel, in *Data Analysis, Machine Learning and Applications: Proceedings of the 31st Annual Conference of the Gesellschaft für Klassifikation e.V., Albert-Ludwigs-Universität Freiburg, March 7–9, 2007*, eds. C. Preisach, H. Burkhardt, L. Schmidt-Thieme and R. Decker, Springer Berlin Heidelberg, Berlin, Heidelberg, 2008, DOI: 10.1007/978-3-540-78246-9_38, pp. 319-326.
10. M. R. C. Berthold, N.; Dill, F.; Gabriel, T. R.; Kötter, T.; Meinl, T.; Ohl, P.; Sieb, C.; Thiel, K.; Wiswedel, B., in *Studies in Classification, Data Analysis, and Knowledge Organization*, Springer, Berlin, 2007.
11. S. Roughley, *Curr Med Chem*, 2018, DOI: 10.2174/0929867325666180904113616.
